# Transcription Factor Repurposing Offers Insights into Evolution of Biosynthetic Gene Cluster Regulation

**DOI:** 10.1128/mBio.01399-21

**Published:** 2021-07-20

**Authors:** Wenjie Wang, Milton Drott, Claudio Greco, Dianiris Luciano-Rosario, Pinmei Wang, Nancy P. Keller

**Affiliations:** a Ocean College, Zhejiang University, Zhoushan, China; b Department of Medical Microbiology and Immunology, University of Wisconsin—Madison, Madison, Wisconsin, USA; c Department of Bacteriology, University of Wisconsin—Madison, Madison, Wisconsin, USA; Universidad de Córdoba

**Keywords:** cross talk, regulatory mechanism, transcription factor, citrinin, xanthocillin, *Aspergillus*, *Penicillium*, evolutionary biology, fungi, secondary metabolism, transcription factors

## Abstract

The fungal kingdom has provided advances in our ability to identify biosynthetic gene clusters (BGCs) and to examine how gene composition of BGCs evolves across species and genera. However, little is known about the evolution of specific BGC regulators that mediate how BGCs produce secondary metabolites (SMs). A bioinformatics search for conservation of the Aspergillus fumigatus xanthocillin BGC revealed an evolutionary trail of *xan-*like BGCs across *Eurotiales* species. Although the critical regulatory and enzymatic genes were conserved in Penicillium expansum, overexpression (OE) of the conserved *xan* BGC transcription factor (TF) gene, *PexanC,* failed to activate the putative *xan* BGC transcription or xanthocillin production in *P. expansum,* in contrast to the role of AfXanC in A. fumigatus. Surprisingly, OE::*PexanC* was instead found to promote citrinin synthesis in *P. expansum* via *trans* induction of the *cit* pathway-specific TF, *ctnA*, as determined by *cit* BGC expression and chemical profiling of *ctnA* deletion and OE::*PexanC* single and double mutants. OE::*AfxanC* results in significant increases of *xan* gene expression and metabolite synthesis in A. fumigatus but had no effect on either xanthocillin or citrinin production in *P. expansum*. Bioinformatics and promoter mutation analysis led to the identification of an AfXanC binding site, 5′-AGTCAGCA-3′, in promoter regions of the A. fumigatus *xan* BGC genes. This motif was not in the *ctnA* promoter, suggesting a different binding site of PeXanC. A compilation of a bioinformatics examination of XanC orthologs and the presence/absence of the 5′-AGTCAGCA-3′ binding motif in *xan* BGCs in multiple Aspergillus and *Penicillium* spp. supports an evolutionary divergence of XanC regulatory targets that we speculate reflects an exaptation event in the *Eurotiales*.

## INTRODUCTION

Filamentous fungi produce numerous secondary metabolites (SMs) that are sources of potent drugs (e.g., penicillin, lovastatin) and harmful mycotoxins (e.g., aflatoxin, patulin) (1–3). The potential pharmaceutical/agrochemical properties of fungal SMs have led to worldwide efforts to sequence, identify, and mine fungal biosynthetic gene clusters (BGCs) that encode SMs (4–7). These studies have revealed hallmark characteristics of BGCs that present obstacles to efficient drug discovery programs. For example, many BGCs are “silent” or “cryptic” under laboratory culture conditions. Sometimes, silent BGCs can be activated through genetic manipulations ranging from heterologous expression in model fungi (8, 9) to epigenetic modification (10, 11) and overexpression/deletion of BGC pathway-specific transcription factors (TFs) (12). However, a large number of BGCs remain silent despite such strategies (8).

A perplexing observation is that some BGCs can be highly expressed in one species or one isolate of a single species but not expressed (i.e., silent or cryptic) in another species or isolate of the same species during growth under typical laboratory conditions, often for no identifiable reason. The aflatoxin BGC presents a salient case of this particularity, where many isolates of Aspergillus flavus have an intact aflatoxin BGC but only some of the strains produce this SM (13). These occurrences suggest that a regulatory mechanism(s) may be responsible for such chemotype differentiation. For example, the aflatoxin BGC contains an in-cluster TF, AflR, which is required to induce expression of the biosynthetic genes (14). Naturally occurring AflR mutations are associated with a loss of aflatoxin production in certain Aspergillus isolates (15, 16). Furthermore, AflR has been found to regulate genes outside of the aflatoxin BGC, suggesting potential of expansion and/or repurposing of BGC TFs (17). This latter concept is supported by several studies. One is the Aspergillus nidulans *inp* BGC TF ScpR, which activates not only in-cluster genes (*inpA* and *inpB*) but also the asperfuranone BGC TF, *afoA* (18). Another example is the trichothecene BGC TF Tri6. In the fungus Trichoderma arundinaceum, this TF regulates expression of both *tri* genes and mevalonate pathway genes, which are required for the synthesis of farnesyl diphosphate (FPP), the primary metabolite that feeds into trichothecene biosynthesis (19).

We recently characterized the first fungal isocyanide BGC, the xanthocillin (*xan*) BGC, in Aspergillus fumigatus (20). The *xan* BGC TF XanC induced expression of *xan* biosynthetic genes and subsequent cluster metabolites in A. fumigatus (20). Xanthocillins have been reported to be synthesized by several Aspergillus and *Penicillium* spp. (21, 22). Despite one report of xanthocillin production by the apple pathogen Penicillium expansum (23) and the presence of the *xan* BGC in this species’ genome (20), we could not detect this metabolite in our chemical profiling studies of a commonly studied *P. expansum* isolate that contained the putative *xan* BGC (24, 25). We hypothesized that a change in *P. expansum* XanC regulatory functionality might explain the lack of metabolite synthesis. In testing this hypothesis, we discovered an unexpected deviation in target gene regulation by *P. expansum* XanC (PeXanC) and A. fumigatus XanC (AfXanC). We found that PeXanC activates only one gene (*PexanG*) in the putative *xan* BGC but, unexpectedly, activates the in-cluster TF, *ctnA*, of the citrinin BGC and, when overexpressed (OE::*PexanC*), leads to high citrinin production in *P. expansum*. No *cit*-like BGC was found in A. fumigatus (3). Placement of the overexpression allele of AfXanC in *P. expansum* did not activate *ctnA* or citrinin production. Analysis of *xan* promoter sequences led to the identification of a conserved, putative AfXanC binding motif (5′-AGTCAGCA-3′) in all A. fumigatus *xan* promoters. Mutation of this site in the A. fumigatus *xanB* promoter significantly decreased *xanB* expression and loss of synthesis of *xan* BGC metabolites. This motif was not in the *ctnA* promoter, suggesting a loss of recognition of this site by PeXanC.

## RESULTS

### A putative *xan* biosynthetic gene cluster in *Penicillium expansum*.

The first fungal isocyanide synthase (ICS)-containing BGCs were described in A. fumigatus, in which one of the ICS BGCs, termed the *xan* BGC, was found to synthesize xanthocillin and its derivatives (20). We identified a putative *xan* BGC, containing five out of seven genes found in A. fumigatus, in *Penicillium expansum* by using MultiGene BLAST (see Fig. S1a in the supplemental material): *PexanA* (PEXP_030240), *PexanB* (PEXP_030230), *PexanC*, *PexanD* (PEXP_030210), and *PexanG* (PEXP_030200). Four of these proteins showed high similarities (identity, >50%; query cover, >85%) with A. fumigatus orthologs, while the putative TF, PeXanC, showed 35.87% identity with query cover of 93% to AfXanC (Fig. S1b). The *P. expansum* BGC contains the two essential enzymes required for xanthocillin synthesis, XanB, an isocyanide synthase-dioxygenase, and XanG, a cytochrome P450 (20, 26). The *P. expansum* cluster lacks orthologs of AfXanE and AfXanF that modify *xan* BGC products through methylation (26). The National Center for Biotechnology Information (NCBI) incorrectly annotated PeXanC as 337 amino acids, which we determined to be 316 amino acids by multiple sequence alignment (Fig. S1c) and gene expression studies. Overexpression of the incorrect *PexanC* gene did not alter production of any metabolite in *P. expansum* (data not shown) in contrast to the results (described below) of overexpression of the correct gene. Furthermore, the incorrect version did not align with the start site of other putative XanC proteins, whereas the correct version did align perfectly.

10.1128/mBio.01399-21.4 FIG S1 Comparison of putative *xan* cluster in *P. expansum* and *xan* cluster in A. fumigatus. (a) Diagram of two *xan* clusters in *P. expansum* and A. fumigatus. Homologous genes are shaded with the same color. (b) Comparison of *xan* genes between putative *xan* cluster in *P. expansum* and *xan* cluster in A. fumigatus. (c) Sequence alignment and nucleotide sequence of PeXanC. Sequence alignment of AfXanC and PeXanC shows the incorrectly predicted initiation methionine of PeXanC (PEXP_030220) from NCBI. The correct protein sequence of PeXanC is indicated with a red frame. The correct nucleotide sequence of *PexanC* is shown below the protein sequence. Download FIG S1, TIF file, 2.2 MB. Copyright © 2021 Wang et al. 2021 Wang et al. https://creativecommons.org/licenses/by/4.0/ This content is distributed under the terms of the Creative Commons Attribution 4.0 International license.

### Overexpression of *PexanC* yields growth phenotypes and results in overproduction of citrinin but does not induce *xan* BGC expression or *xan* metabolites.

As *P. expansum* contains *PexanB* and *PexanG*, we hypothesized that this fungus has the capability of producing xanthocillin despite our inability to detect the metabolite previously (24, 25) and thus reasoned that overexpression of *PexanC* should induce xanthocillin synthesis. By following the strategy used to characterize the A. fumigatus *xan* metabolites, we both deleted and overexpressed *PexanC*, expecting to see overexpression of *Pexan* BGC gene expression and consequent metabolite production. First, a *pyrG^−^* auxotrophic strain TDL9.1 was created from *P. expansum* strain TJT14.1 (27) to allow use of *pyrG* as a selective marker for the creation of subsequent strains. TDL9.1 was restored to prototrophy to create the *pyrG* complemented strain, TDL12.1, as the *P. expansum* control strain. *PexanC* was overexpressed using the constitutive promoter *gpdAp* from Aspergillus nidulans (28). Three OE::*PexanC* strains, TWW4.1, TWW4.2, and TWW4.3, were used for subsequent experimentation. The *ΔPexanC* strain, TWW17.1, was created by deleting *PexanC* and replacing it with A. fumigatus *pyrG*. All transformants were confirmed by PCR and Southern blotting (Fig. S2) with construction details provided in Materials and Methods.

10.1128/mBio.01399-21.5 FIG S2 DNA constructs and Southern blot confirmation. (a to i) DNA construct and digestion strategy of the *ΔpyrG* mutant (TDL9.1), *pyrG* complemented strain (TDL12.1), OE::*PexanC* mutant (TWW4), *ΔPexanC* mutant (TWW17.1), *ΔctnA* mutant (TWW19.1), *ΔctnA* OE::*PexanC* double mutant (TWW18.1), OE::*AfxanC-Pe* mutant (TWW5.1), BSm OE::*PexanC* double mutant (TWW31.1), BSc OE::*PexanC* double mutant (TWW32.1), and *Δ*motif OE::*PexanC* double mutant (TWW27.1) carried out with the indicated restriction enzymes, and Southern blot analysis showing predicted DNA fragments. Both left and right flanking sequences of the target gene were used as probes. Download FIG S2, TIF file, 0.6 MB. Copyright © 2021 Wang et al. 2021 Wang et al. https://creativecommons.org/licenses/by/4.0/ This content is distributed under the terms of the Creative Commons Attribution 4.0 International license.

**FIG 1:**
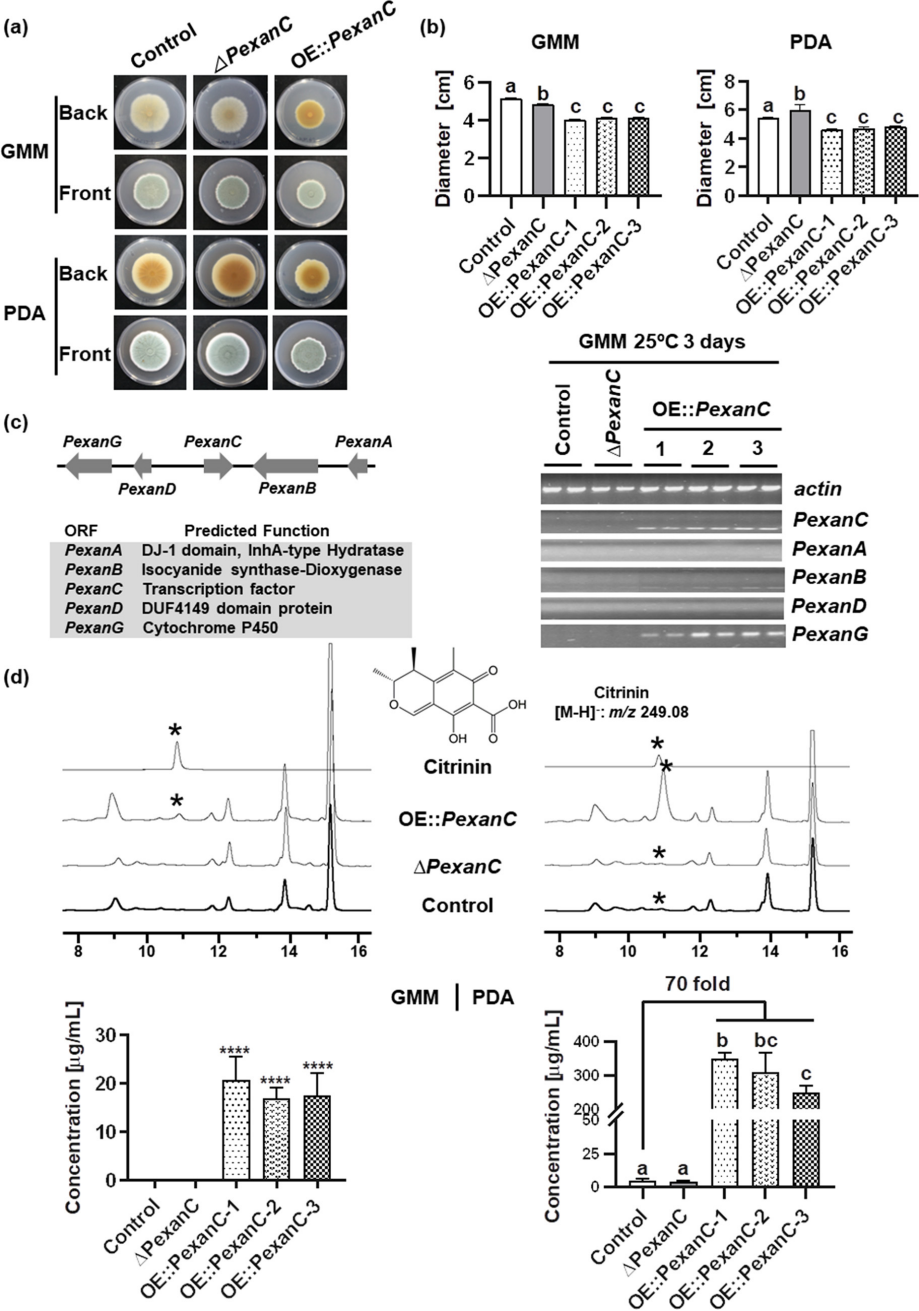
Overexpression of *PexanC* decreases colony diameter and increases the production of citrinin instead of any product from the *xan* cluster in *P. expansum*. (a) The phenotypes of control, *ΔPexanC*, and OE::*PexanC* strains on GMM and PDA plates after 14 days of inoculation at 25°C, grown in the dark. (b) Colony diameters of control, *ΔPexanC*, and OE::*PexanC* strains on GMM and PDA plates from panel a. (c) Semiquantitative PCR shows the expression of *Pexan* genes in control, *ΔPexanC*, and three OE::*PexanC* strains grown on GMM at 25°C for 3 days. The predicted functions of *Pexan* genes are listed. (d) Comparative metabolomics of *P. expansum* control, *ΔPexanC*, and OE::*PexanC* strains with the citrinin standard. Asterisks denote the citrinin peak. The chemical structure of citrinin is shown. The production of citrinin in all strains was quantified based on the standard curve (Data Set S1). One-way ANOVA differences were considered significant when the *P *value was <0.05. Different letters above the columns indicate statistically significant differences between the strains as determined using Tukey’s single-step multiple comparison test; concentrations are the mean and standard deviation (SD) of four replicates. ****, *P *< 0.0001.

The control (TDL12.1), OE::*PexanC*, and *ΔPexanC* strains were compared for growth characteristics and chemical profiles. All strains were grown on both glucose minimal medium (GMM) and potato dextrose agar (PDA) solid media. All three OE::*PexanC* strains showed decreased diameter growth and produced a distinct yellow pigment on the bottom of GMM plates compared to the control and *ΔPexanC* strains (Fig. 1a and b). In PDA medium, all strains produced a brown pigment and again all OE::*PexanC* strains were reduced in diameter growth compared to the other strains. The *ΔPexanC* strain had a larger diameter growth than the control strain (Fig. 1a and b).

10.1128/mBio.01399-21.8 DATA SET S1 Citrinin structure and UV spectrum, and production of citrinin of strains in GMM and PDA with the standard curve of citrinin. Download Data Set S1, XLSX file, 0.05 MB. Copyright © 2021 Wang et al. 2021 Wang et al. https://creativecommons.org/licenses/by/4.0/ This content is distributed under the terms of the Creative Commons Attribution 4.0 International license.

The expression of *Pexan* genes was examined by semiquantitative PCR (semi-qPCR) in GMM (Fig. 1c). None of the *Pexan* genes were expressed in either the control or *ΔPexanC* strains, confirming that this cluster was silent under standard laboratory conditions. Unexpectedly, OE::*PexanC* induced only *PexanG* expression but not expression of the isocyanide synthase gene *PexanB* or other *xan* genes. Matching these transcriptional data, none of the OE::*PexanC* strains produced any xanthocillin or xanthocillin-like isocyanides on GMM or PDA. However, when the strains were grown on GMM, we could detect a new peak at ∼11 min from the OE::*PexanC* strain that was not detected in the control or *ΔPexanC* strains (Fig. 1d). This peak showed two ions in electrospray ionization (ESI) negative mode, *m/z* 249.07646 and *m/z* 267.08762 with maximum absorption wavelengths of 215 nm and 330 nm in the UV spectrum (Fig. 1d and Data Set S1) and with predicted molecular formulas C_13_H_13_O_5_^-^ and C_13_H_15_O_6_^-^. Mass and UV spectrum data were in accordance with those of citrinin (29–31), a compound known to be produced by *P. expansum* in citrinin-producing medium (27). This peak was confirmed to be citrinin by comparison to 10 μl of citrinin standard (0.1 mg/ml) (Fig. 1d), which showed the same two ions, *m/z* 249.08 [M − H]^−^ and *m/z* 267.09 [M + H_2_O − H]^−^. PDA extracts showed the presence of citrinin in all strains (control, *ΔPexanC*, and OE::*PexanC* strains) but with a 70-fold increase in OE::*PexanC* strains (Fig. 1d and Data Set S1).

10.1128/mBio.01399-21.7 FIG S4 Putative motif was confirmed not to be the real PeXanC binding site. (a) Conserved motif for putative PeXanC binding site. The motif was searched by MEME (https://meme-suite.org). (b) The predicted motif (5′-TGGNTGNG-3′) is confirmed not to be the DNA binding site of PeXanC. Semi-qPCR result for *ctnA* and *citS* expression in TWW27.1 (*Δ*motif OE::*PexanC*) shows no difference from that of the OE::*PexanC* strain. Download FIG S4, TIF file, 0.4 MB. Copyright © 2021 Wang et al. 2021 Wang et al. https://creativecommons.org/licenses/by/4.0/ This content is distributed under the terms of the Creative Commons Attribution 4.0 International license.

### PeXanC regulates the citrinin BGC through activation of the in-cluster transcription factor *ctnA*.

**FIG 2:**
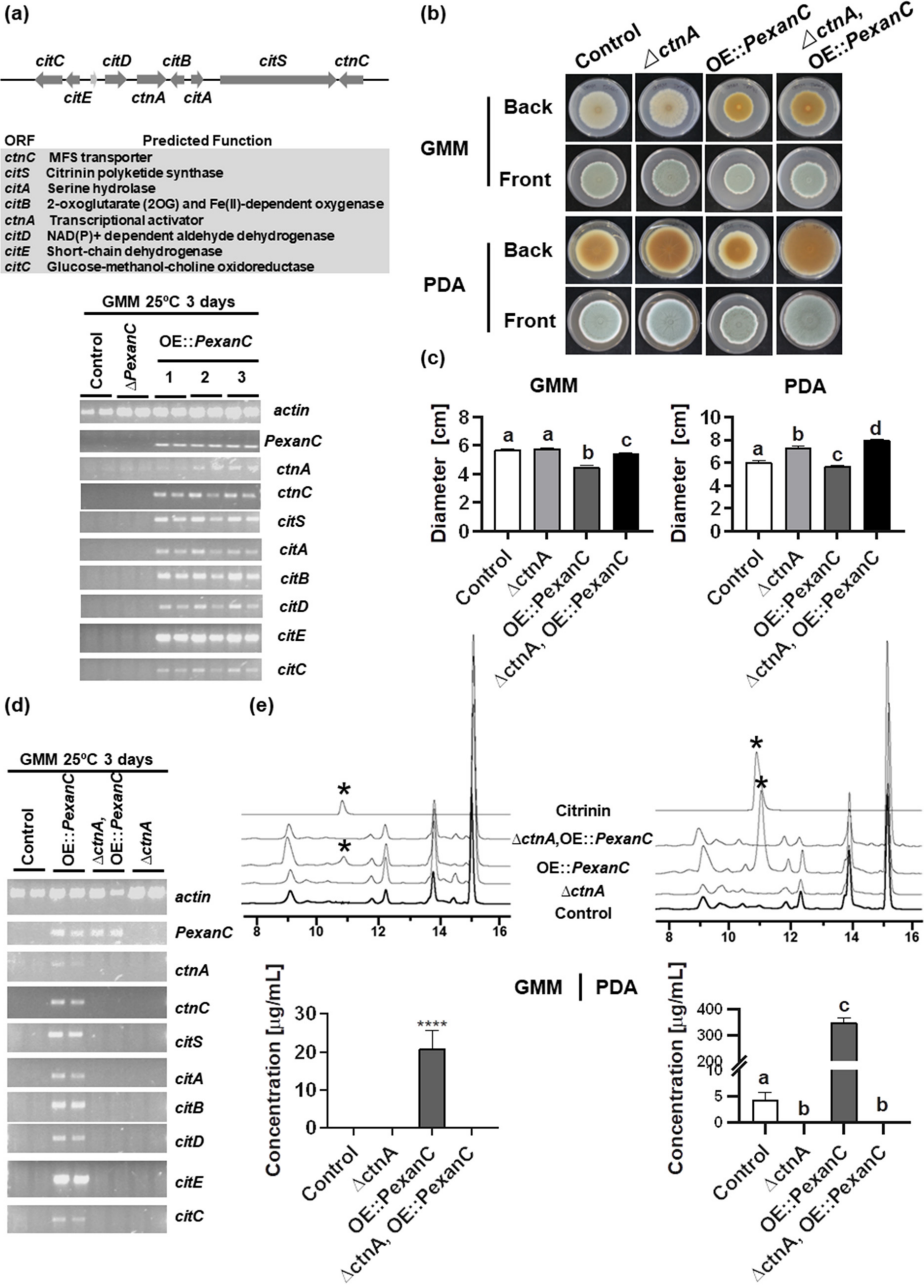
*PexanC* regulates the *cit* cluster through its TF *ctnA* gene. (a) Semiquantitative PCR shows the gene expression of *cit* genes in control, *ΔPexanC*, and three OE::*PexanC* strains on GMM at 25°C for 3 days. The predicted functions of *cit* genes are listed. (b) The phenotypes of control, *ΔctnA*, OE::*PexanC*, and *ΔctnA* OE::*PexanC* double mutant strains on GMM and PDA plates after 14 days inoculation at 25°C, under dark conditions. (c) Colony diameters of control, *ΔctnA*, OE::*PexanC*, and *ΔctnA* OE::*PexanC* double mutant strains on GMM and PDA plates from panel a. (d) Semiquantitative PCR shows expression of *cit* genes of the control, *ΔctnA*, OE::*PexanC*, and *ΔctnA* OE::*PexanC* double mutant strains grown on GMM at 25°C for 3 days. (e) Comparative metabolomics of *P. expansum* control, *ΔctnA*, OE::*PexanC*, and *ΔctnA* OE::*PexanC* double mutant strains with the citrinin standard. Asterisks denote the citrinin peak. The production of citrinin in control, *ΔctnA*, OE::*PexanC*, and *ΔctnA* OE::*PexanC* double mutant strains was quantified by the standard curve (Data Set S1). One-way ANOVA differences were considered significant when the *P* value was  <0.05. Different letters above the columns indicate statistically significant differences between the strains as determined using Tukey’s single-step multiple comparison test; concentrations shown are the mean and SD of four replicates. ****, *P *< 0.0001.

Gene expression and chemical profiles (Fig. 1c and d) suggested that PeXanC activated the citrinin (*cit*) BGC rather than the *Pexan* BGC. Using the same conditions as for *xan* gene expression (Fig. 1c), we next assessed *cit* BGC expression in the OE::*PexanC* strains compared to the control and *ΔPexanC* strains. As shown in Fig. 2a, all of the *cit* genes were highly expressed in the three OE::*PexanC* strains.

The *cit* BGC includes an in-cluster Zn(II)_2_Cys_6_ transcription factor named CtnA (or Mrl3 in Monascus ruber M7 [30]), which is thought, but not reported, to activate expression of the biosynthetic *cit* genes (30, 32). To determine if PeXanC induced *cit* gene expression via CtnA, the *ctnA* gene was deleted in both the control strain and one OE::*PexanC* strain (TWW4.1) to create the *ΔctnA* strain (TWW19.1) and the *ΔctnA* OE::*PexanC* double mutant (TWW18.1). All mutants were confirmed by PCR and Southern blotting (Fig. S2).

Both the *ΔctnA* strain and the *ΔctnA* OE::*PexanC* double mutant were grown on GMM and PDA solid media concurrently with the respective control strain and the parental OE::*PexanC* strain. On GMM, the *ΔctnA* mutant and control strain grew similarly. In contrast, deletion of *ctnA* in the OE::*PexanC* background partially remediated the growth defect of the OE::*PexanC* single mutant strain (Fig. 2b and c). The effect of *ctnA* loss was more dramatic on PDA medium, where the *ΔctnA* and *ΔctnA* OE::*PexanC* strains were observed to have an increased colony diameter compared with that of the control and OE::*PexanC* strains, respectively (Fig. 2b and c).

Expression of *cit* genes was assessed by semi-qPCR in GMM (Fig. 2d). No *cit* gene was expressed in either the control or *ΔctnA* strains. As shown above (Fig. 2a), all *cit* genes were activated in the OE::*PexanC* strain but not in the *ΔctnA* OE::*PexanC* double mutant. Concomitantly, citrinin was not produced by any *ctnA* deletion strain in either GMM or PDA (Fig. 2e and Data Set S1). This indicates that PeXanC regulates the *cit* cluster through CtnA and CtnA appears to be essential for activating the remaining *cit* BGC genes to produce citrinin (Fig. 2d).

### DNA binding site recognition contributes to differences in target gene regulation by PeXanC and AfXanC.

**FIG 3:**
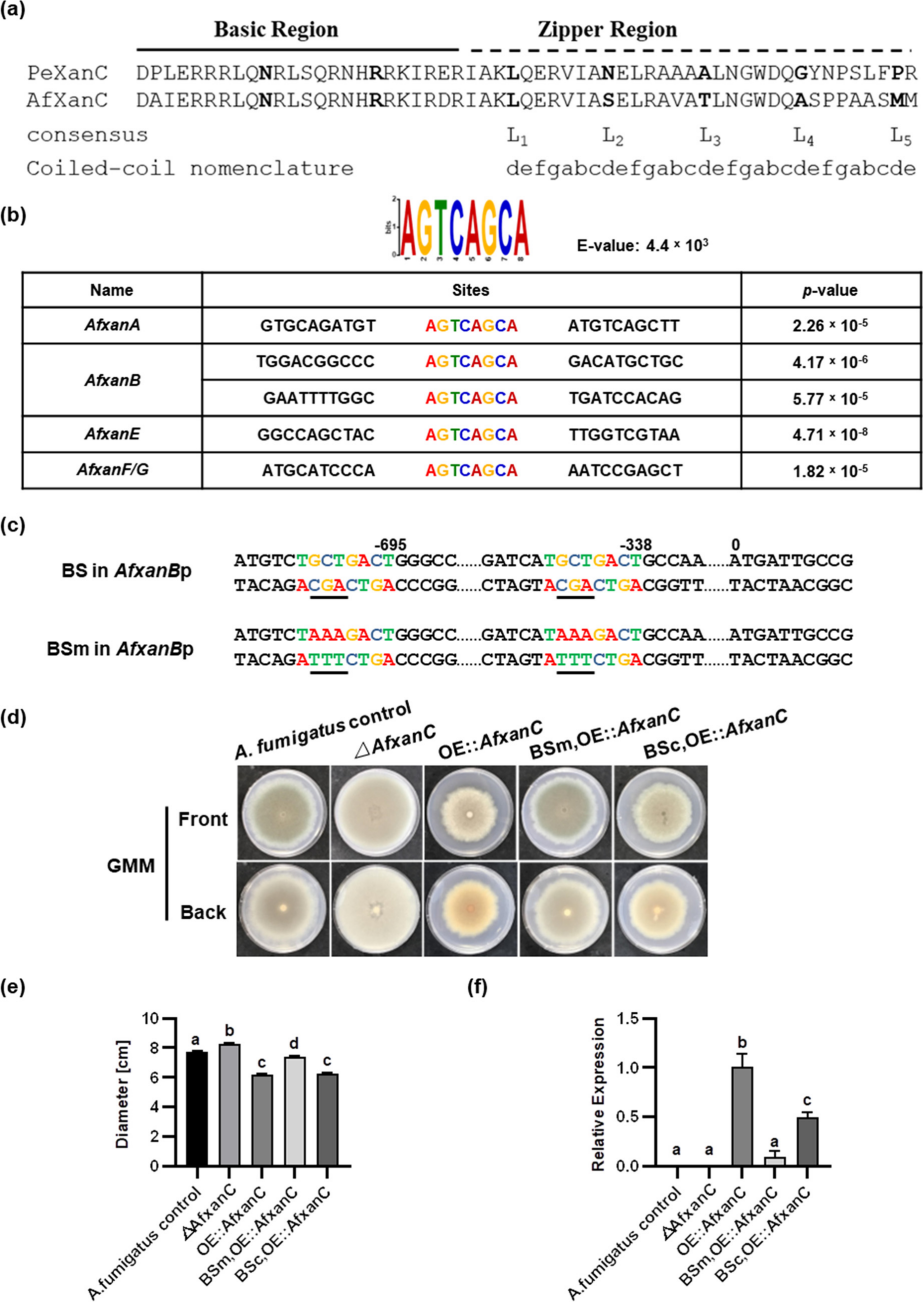

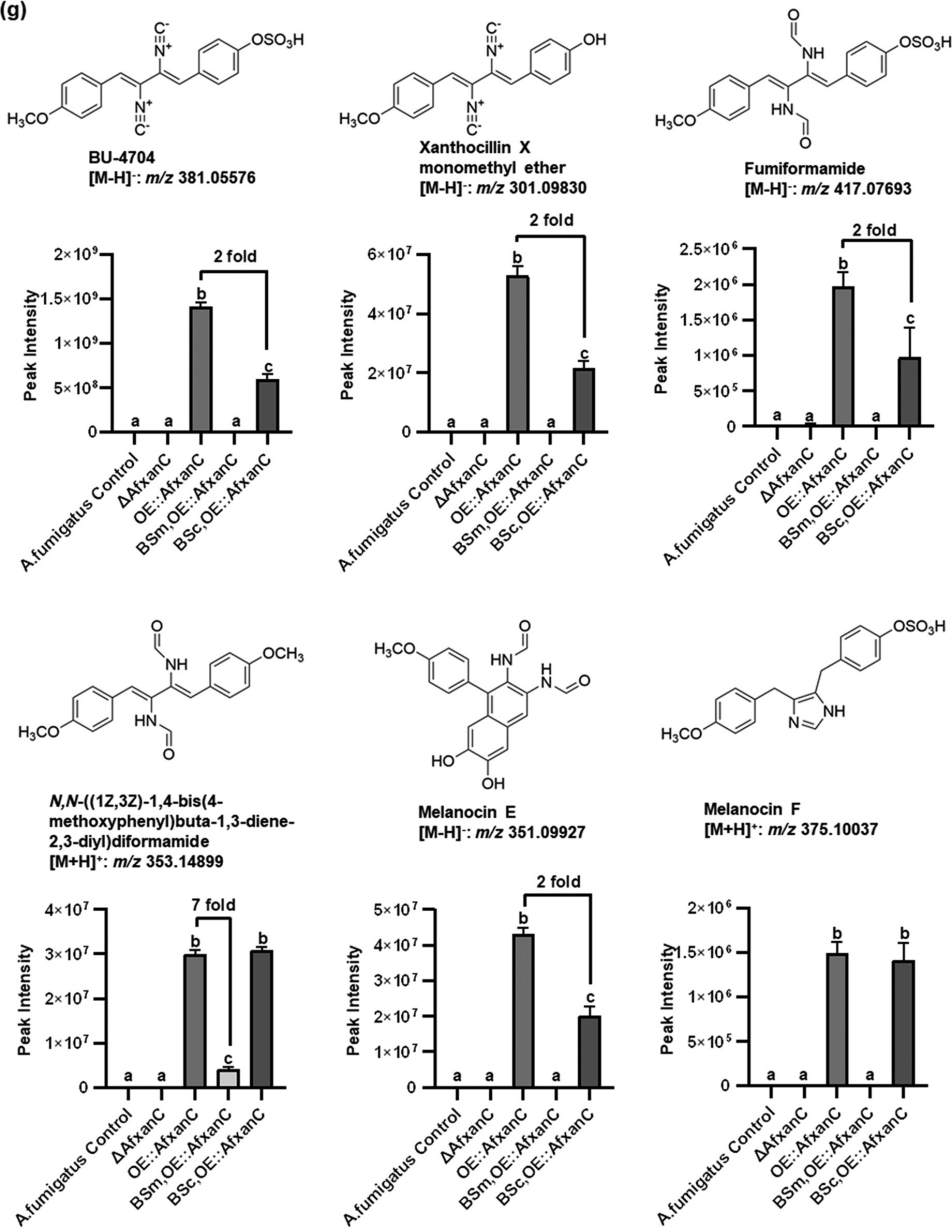
DNA binding site of AfXanC is confirmed to be 5′-AGTCAGCA-3′. (a) bZIP conserved domain (basic region and zipper region) in PeXanC and AfXanC. The characterized amino acids are in bold, comprising two amino acids in the basic region and five amino acids in the zipper region supposed to be leucine residues. (b) The 5′-AGTCAGCA-3′ motif was identified using MEME and is shown with a summary of the promoter sites for the *Afxan* genes. (c) Mutagenesis of two binding sites, 5′-AGTCAGCA-3′ to 5′-AGTCTTTA-3′, in the *AfxanB* promoter. BS, binding site; BSm, binding site mutant. (d) The phenotypes of A. fumigatus control, *ΔAfxanC*, OE::*AfxanC*, and BSm OE::*AfxanC* and BSc OE::*AfxanC* double mutant strains on GMM after 7 days of inoculation at 37°C, under dark conditions. (e) Colony diameters of all the strains on GMM from panel d. (f) qPCR shows relative gene expression of *AfxanB* in A. fumigatus control, *ΔAfxanC*, and BSm OE::*AfxanC* and BSc OE::*AfxanC* double mutant strains compared to that of the OE::*AfxanC* strain. (g) Production of xanthocillin derivatives in all of these A. fumigatus strains. One-way ANOVA differences were considered significant when the *P* value was <0.05. Different letters above the columns indicate statistically significant differences between the strains as determined using Tukey’s single-step multiple comparison test; values shown are the mean and SD of four replicates.

TFs regulate genes by binding to specific DNA binding sites in the promoter region of target genes (33). We considered that variation in binding site motif recognition by the two XanC TFs and/or loss/gain of binding sites in promoters might explain the differential regulation by PeXanC and AfXanC. Both orthologs are annotated as bZIP proteins, which are characterized by a conserved basic region and a more diversified leucine zipper motif (34, 35). Examination of PeXanC and AfXanC showed a conservation of key residues in the basic region, but neither protein contained the typical leucine residues in their zipper region (Fig. 3a and Fig. S1c).

Typically, in-cluster BGC TFs recognize a conserved motif in the promoters of the BGC gene members, such as AflR’s recognition of 5′-TCG(N5)CGA-3′ in multiple *stc* BGC genes (36). Since AfXanC activates all *xan* genes in A. fumigatus (20), we reasoned that its binding site would be conserved in *xan* promoters. We examined all A. fumigatus *xan* promoters by multiple expectation maximizations for motif elicitation (MEME), an algorithm to identify related DNA motifs (37) (https://meme-suite.org). A conserved motif (5′-AGTCAGCA-3′) was found with an E value of 4.4 × 10^3^ (Fig. 3b). This motif was found in all the promoters of A. fumigatus *xan* genes (*xanA*, *xanB*, *xanE*, *xanF*, *xanG*) regulated by AfXanC but not in the promoters of *xanC* itself or *xanD*, which was not regulated by XanC (20) and did not contribute to product formation (26).

To confirm the functionality of the predicted AfXanC recognition site, a binding site mutant (BSm OE::*AfxanC*; TWW31.1) was made by mutating the 5′-AGTCAGCA-3′ motif into 5′-AGTCTTTA-3′ in the promoter region of *AfxanB* in an OE::*AfxanC* background in A. fumigatus (Fig. 3c). To eliminate the possible effect of the selective marker, *argB*, which was used in the mutant construction, a binding site control strain (BSc OE::*AfxanC*; TWW32.1) was made with *argB* inserted in the same location while leaving the binding site intact. All mutants were confirmed by PCR and Southern blotting (Fig. S2).

Both TWW31.1 and TWW32.1 were grown in GMM, and their gross colony phenotype, *AfxanB* gene expression, and *xan* BGC product titer were compared to those of the A. fumigatus *xan* BGC wild-type (WT) strain (TFYL81.5) (26, 38) and the previously characterized *ΔAfxanC* (TNLR9.1) and OE::*AfxanC* (TNLR1.2) strains (20, 26). Consistent with the previous data (20), the OE::*AfxanC* strain showed a decreased colony diameter and accumulation of a yellow pigment at the bottom of the plate in comparison to the control and *ΔAfxanC* strains (Fig. 3d and e). The growth of the binding site control strain (BSc OE::*AfxanC*) was similar to that of the OE::*AfxanC* strain but presented a decreased colony diameter and some yellow pigment on the colony underside relative to the control and *ΔAfxanC* strains. However, the growth of the binding site mutant (BSm OE::*AfxanC*) was nearly equivalent to the phenotype of the WT control strain (Fig. 3d and e).

*AfxanB* expression was then quantified by qPCR. As previously mentioned, both the WT control and *ΔAfxanC* strains do not express *AfxanB* under normal culture conditions (GMM medium) (20). However, as expected, *AfxanB* was highly expressed in the OE::*AfxanC* strain (Fig. 3f). Compared to the OE::*AfxanC* strain, the binding site mutant showed a >90% decrease (*P* < 0.0001) in *AfxanB* expression, which was not statistically different from that of the WT (*P *= 0.4814) and *ΔAfxanC* (*P *= 0.4367) strains (Fig. 3f). In contrast, *AfxanB* expression was only ∼50% decreased (*P* < 0.0001) in the binding site control strain in comparison to the OE::*AfxanC* strain (Fig. 3f).

Chemical profile results of *xan* BGC metabolites recapitulated *xanB* gene expression in the five strains (A. fumigatus WT control, *ΔAfxanC*, and OE::*AfxanC* strains and BSm OE::*AfxanC* and BSc OE::*AfxanC* double mutants) (Data Set S2). No metabolites were observed in the WT control and *ΔAfxanC* strains (Fig. 3g), and of the six quantifiable *xan* BGC products produced by the OE::*AfxanC* strain [BU-4705, xanthocillin X monomethyl ether, fumiformamide, *N*,*N*-((1*Z*,3*Z*)-1,4-bis(4-methoxyphenyl)buta-1,3-diene-2,3-diyl)diformamide, melanocin E, and melanocin F] (20, 26), only a small amount of *N*,*N*-((1*Z*,3*Z*)-1,4-bis(4-methoxyphenyl)buta-1,3-diene-2,3-diyl)diformamide was detected in extracts of the binding site mutant. In contrast, two metabolites [*N*,*N*-((1*Z*,3*Z*)-1,4-bis(4-methoxyphenyl)buta-1,3-diene-2,3-diyl)diformamide and melanocin F] were produced by the binding site control at the same level (*P *= 0.1620, *P *= 0.7921), and the other four metabolites were reduced by approximately 50% (*P* < 0.0001) compared to the OE::*AfxanC* strain (Fig. 3g). Taken together, these data support 5′-AGTCAGCA-3′ as an AfXanC binding site.

10.1128/mBio.01399-21.9 DATA SET S2 Production of xanthocillin derivatives in binding site mutant and other A. fumigatus strains. Download Data Set S2, XLSX file, 0.01 MB. Copyright © 2021 Wang et al. 2021 Wang et al. https://creativecommons.org/licenses/by/4.0/ This content is distributed under the terms of the Creative Commons Attribution 4.0 International license.

**FIG 4:**
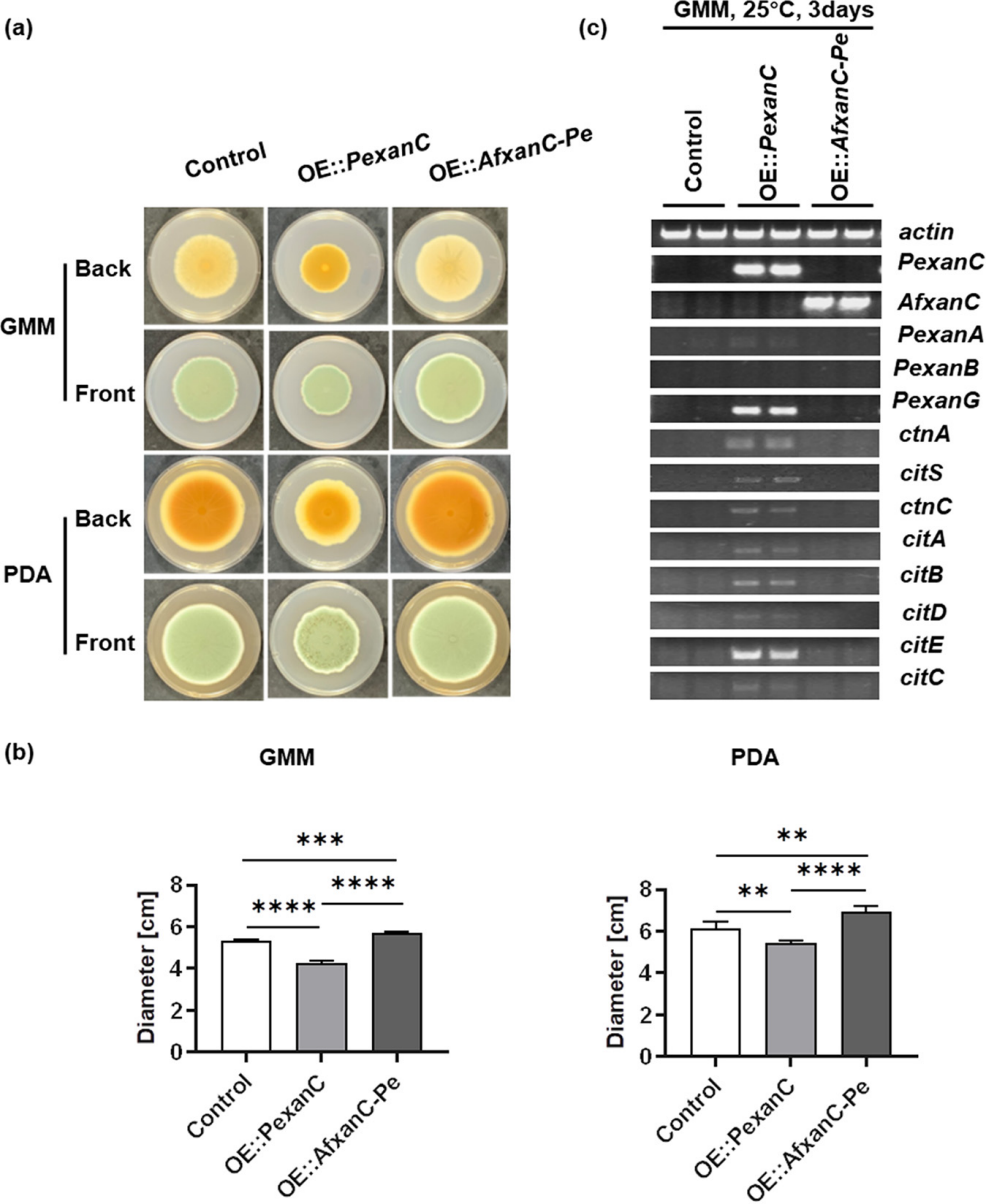
Overexpression of *AfxanC* in *P. expansum* could not activate any *Pexan* or *cit* genes. (a) Phenotypes of *P. expansum* control, OE::*PexanC*, and OE::*AfxanC-Pe* strains on GMM and PDA after 14 days of inoculation at 25°C, under dark conditions. (b) Colony diameters of all strains from panel a. **, 0.0002 < *P* < 0.0021; ***, 0.0001 < *P* < 0.0002; ****, *P* < 0.0001. (c) Semi-qPCR shows expression of *Pexan* genes and *cit* genes of the control, OE::*PexanC*, and OE::*AfxanC-Pe* strains grown on GMM at 25°C for 3 days.

Examination of *Pexan* and *cit* gene promoters found only one 5′-AGTCAGCA-3′ site in *xan* BGC promoters (i.e., the *PexanA* promoter) and no sites in the *ctnA* or other *cit* gene promoters (Table S1), suggesting that PeXanC preferentially recognizes a different binding site than AfXanC. To examine this hypothesis further, we placed an OE::*AfxanC* allele into *P. expansum* by replacing the *PexanC* gene to create strain TWW5.1 (OE::*AfxanC-Pe*). The *P. expansum* control, OE::*PexanC*, and OE::*AfxanC-Pe* strains were then grown on GMM and PDA solid media. In contrast to the poorer growth of the OE::*PexanC* strain, the growth of the OE::*AfxanC-Pe* strain is similar to that of the control strain (Fig. 4a and b). *xan* and *cit* expression was then analyzed by semi-qPCR (Fig. 4c). No *Pexan* genes were expressed in the OE::*AfxanC-Pe* strain, suggesting that AfXanC could not recognize the motif in the *PexanA* promoter. The inability of AfXanC to promote *PexanA* expression could be due to a number of reasons, such as a requirement for further residues in addition to 5′-AGTCAGCA-3′ for AfXanC binding and/or a requirement for additional transcriptional elements that are not present in the *P. expansum* genome, such as a binding partner for AfXanC. AfXanC also did not regulate *ctnA* or any *cit* genes. Taken together, these results suggest separate regulatory pathways for AfXanC and PeXanC.

10.1128/mBio.01399-21.1 TABLE S1 Search of AfXanC binding site 5′-AGTCAGCA-3′ in the promoters of *xan* genes in the *Eurotiales* spp. and specifically in *Pexan* genes and *cit* genes in *P. expansum*. Download Table S1, PDF file, 0.3 MB. Copyright © 2021 Wang et al. 2021 Wang et al. https://creativecommons.org/licenses/by/4.0/ This content is distributed under the terms of the Creative Commons Attribution 4.0 International license.

### Evolutionary considerations of XanC regulation from analysis of *xan* and *cit* gene cluster families.

Although our experimental data supported 5′-AGTCAGCA-3′ as an AfXanC binding site, we were unable to predict a PeXanC binding site, since the number of target genes (*PexanG* and *ctnA*) was too small for MEME analysis. To gain insight into a possible PeXanC binding site and to gain perspective on the evolution of XanC in fungi, we identified homologous *xan* and *cit* BGCs using multigene BLASTN and BLASTP (see Materials and Methods for details). Because recent analysis of gene cluster families (GCFs) across all fungi (39) has suggested that the *cit* GCF is narrowly distributed within *Eurotiales*, we limited our search to the NCBI genome database within this group. In order to avoid possibly pseudogenized cluster variants that might confound our assessment of the relationship between *xanC* sequences and the presence of the *xan* and *cit* BGCs, we selected 16 species (11 *Penicillium* spp. and 5 Aspergillus spp.) (Fig. S3a) based on highly conserved *xan* or *xan*-like BGCs (all containing *xanB* and *xanG*) or previous reports of xanthocillin production (22, 40). Additionally, we selected 10 species (including 4 *Penicillium* spp., 5 Aspergillus spp., and 1 *Monascus* spp.) (Fig. S3a) found to contain putative *cit* or *cit-*like BGCs based on similar criteria (detailed further in Materials and Methods). Three species, *P. expansum*, Penicillium ferii, and A. nidulans, possessed both BGCs.

10.1128/mBio.01399-21.6 FIG S3 Bioinformatics analyses of XanC in *Eurotiales* spp. (a) Genome accession numbers for *Eurotiales* species used to establish a relationship between the *xanC* sequence and the presence/absence of xanthocillin and citrinin BGCs. (b) Alignment of XanC homologs in Aspergillus spp. and *Penicillium* spp. Download FIG S3, TIF file, 2.3 MB. Copyright © 2021 Wang et al. 2021 Wang et al. https://creativecommons.org/licenses/by/4.0/ This content is distributed under the terms of the Creative Commons Attribution 4.0 International license.

**FIG 5:**
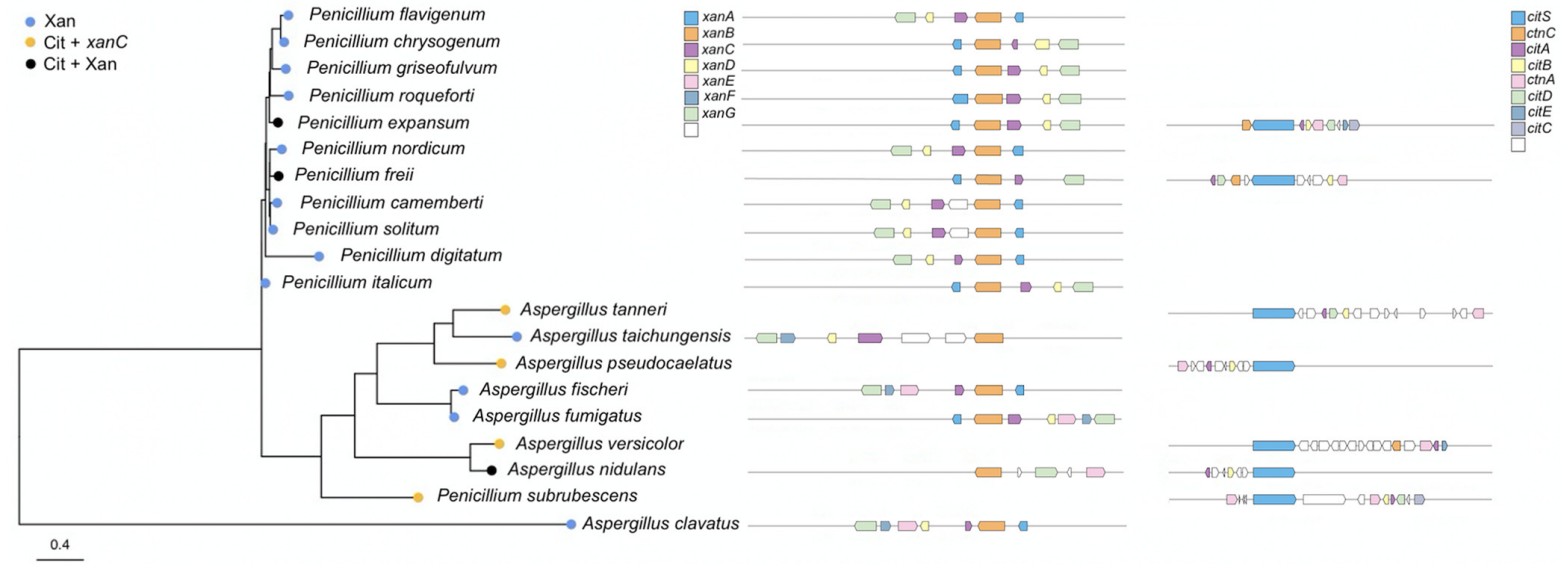
Distribution of *xan* and *cit* cluster in *Eurotiales* compared to XanC maximum likelihood phylogeny. Strains that contain only a putative *xan* cluster are indicated by blue dots, while strains that contain a putative *cit* cluster with a single *xanC* gene are indicated by yellow dots, and strains containing both a putative *xan* cluster and *cit* cluster are indicated by black dots. Three strains (*Monascus purpureus*, Penicillium citrinum, *Penicillium verrucosum*) do not have a putative *xanC* gene. Putative *xan* clusters are depicted in the center, and putative *cit* clusters are depicted on the right.

XanC homologs were identified in species with BGCs of interest based on reciprocal best hit BLAST (see alignment of XanC homologs in Fig. S3b). While this approach made it possible to differentiate XanC homologs from similar regulatory genes, it did preclude analysis of three genomes (Monascus purpureus, Penicillium citrinum, and Penicillium verrucosum) for which no annotation was available. In genomes in which XanC was present, it was almost always found in a *xan*-like BGC, with the notable exception of A. nidulans, where a putative XanC (∼30% protein identity) was located in isolation on a different chromosome from the *xan*-like BGC in this fungus (Fig. 5). Four species (Aspergillus tanneri, Aspergillus pseudocaelatus, Aspergillus versicolor, and Penicillium subrubescens) containing only a *cit* or *cit-*like BGC also contained a putative XanC protein that, as with A. nidulans, was not found in a BGC. We found no clear association between the presence of xanthocillin and/or citrinin BGCs and XanC protein sequence (see the phylogenetic tree built with the XanC protein sequence in Fig. 5).

We assessed 77 promoters from all putative *xan* genes to look for the presence of the AfXanC binding site (5′-AGTCAGCA-3′). Twenty-four binding sites were found in 22 *xan* promoters (Table S1). Interestingly, Aspergillus fischeri, which is close to A. fumigatus in the phylogenetic tree, also had this binding site in all the promoters of *xan* genes, with two binding sites in the promoter of *xanB*, showing exactly the same number and location as in the A. fumigatus *xan* BGC. In Aspergillus clavatus, the binding site was found in three *xan* genes (*xanA*, *xanE*, *xanG*) but not in *xanB*. We could not find this binding site in any *xan* genes in A. nidulans, which has *xanB*, *xanE*, *xanG*, and an out-of-cluster *xanC*. We found the AfXanC binding site at a much lower frequency in *Penicillium* spp. (11 motifs out of 54 promoters) than in Aspergillus spp. (13 motifs out of 23 promoters). Interestingly 9/11 binding sites found in *Penicillium* spp. occurred in the promoter region of *xanA*. Across all species queried, only A. fumigatus and the closely related A. fischeri possessed a binding motif in the *xanB* promoter region (Table S1). We speculate that these data may reflect the evolution of differential XanC regulatory targets between *Eurotiales* spp.

Using the same strategy, we collected promoter regions from *PexanG* and 9 putative *ctnA* genes. MEME analysis of these 10 promoters resulted in a predicted DNA binding motif (5′-TGGNTGNG-3′) with an E value of 3.9 × 10^3^ (Fig. S4a). However, when we created a *P. expansum* mutant (TWW27.1) where this sequence was deleted in the *ctnA* promoter in an OE::*PexanC* background, *ctnA* and *citS* expression remained equivalent to that of the OE::*PexanC* strain (Fig. S4b), indicating that this was not the PeXanC binding site.

## DISCUSSION

The prospect of mining fungal genomes for lucrative pharmaceuticals/agrochemicals as well as concern about fungal toxins as virulence factors/food contaminants has led to in-depth studies of fungal secondary metabolism. Bioinformatic algorithms coupled with molecular technology have greatly advanced identification of biosynthetic gene clusters (BGCs) and their chemical products (10, 12, 28, 41, 42). These studies have also provided insight into gene composition of BGCs and the evolution of BGCs across species and genera (42, 43). However, despite the need for and interest in exploring endogenous regulation of BGCs, there is still a gap in understanding how BGC transcription factors (TFs) regulate BGCs. Confounding studies include the finding that not all BGCs contain in-cluster TFs (44, 45) and that some in-cluster TFs are nonfunctional (15, 16) or regulate genes outside of the BGC (17, 18). Our work here provides an advance in understanding how TF regulation can evolve and drive BGC regulation in fungal species.

**FIG 6:**
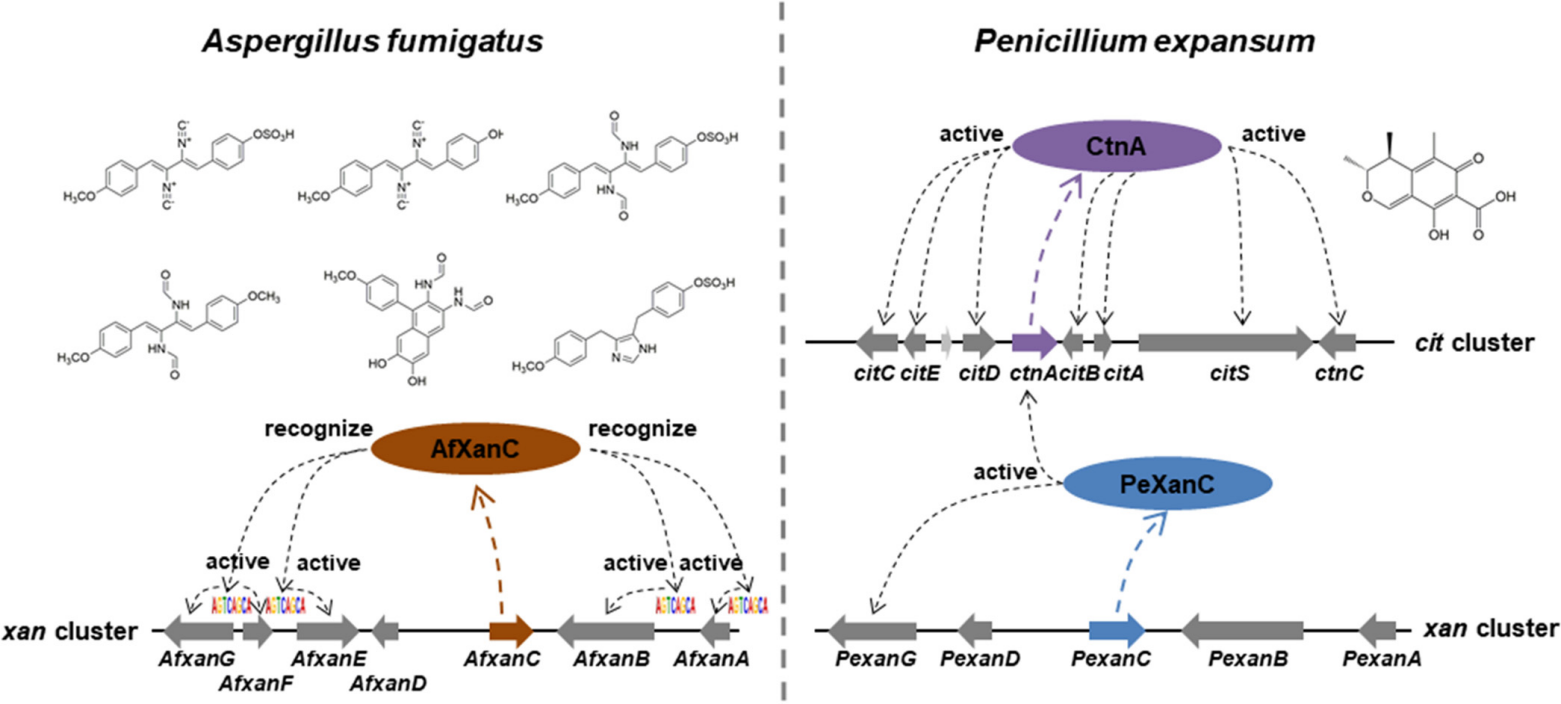
AfXanC and PeXanC have distinct regulatory profiles in A. fumigatus and *P. expansum*.

We found that a primary underlying difference in regulation by AfXanC and PeXanC of the *xan* BGC and *cit* BGC rests in the difference of their recognition of motifs found in promoters (Fig. 6). All of the A. fumigatus *xan* promoters contained the 5′-AGTCAGCA-3′ motif. Mutation of this site in the *AfxanB* promoter reduced *AfxanB* expression by 90.4% and almost entirely eliminated synthesis of any *xan* BGC metabolite by A. fumigatus (Fig. 3g). This motif was found only in the promoter of *xanA* in *P. expansum*. Overexpression of A. fumigatus *xanC* in *P. expansum* did not result in expression of *PexanA* or other *xan* genes (Fig. 4c). A larger as yet unknown AfXanC recognition motif and/or a requirement for additional transcriptional elements not present in *P. expansum* might be one reason for the inability of AfXanC to activate *PexanA*. AfXanC also did not regulate the *cit* BGC (which does not contain a 5′-AGTCAGCA-3′ motif), but this BGC is positively regulated by PeXanC via CtnA activation (Fig. 6). Taken together, these results suggest that PeXanC recognizes a different binding motif than AfXanC. Whereas it is possible that PeXanC regulation of *ctnA* is not direct, the pattern of regulation of another BGC is reminiscent of *trans* regulation of the asperfuranone TF by the *inp* BGC TF ScpR in A. nidulans (18).

Since *PexanC* is physically linked to other genes in the putative *P. expansum xan* BGC, we speculate that the ancestral function of this gene is to regulate the *xan*-like BGC in *P. expansum*. It is possible that an ancestral *xanC* evolved in a genome that contained both the *xan* and *cit* BGCs or that this gene evolved in isolation from the *cit* BGC. The varied distribution of *xan* and *cit* BGCs across our sample of *Eurotiales* (Fig. 5) offers little insight to differentiate these hypotheses. Indeed, our phylogeny of *xanC* sequences mirrors taxonomic relationships between isolates with no other clear associations. We speculate that the Aspergillus clade of the XanC tree (Fig. 5) might also reflect that the DNA-binding motif that is important in the regulatory action of AfXanC is more common in these species. A growing body of work has shown that the regulation of secondary metabolite production can be mediated by ecological interactions (46). Our work demonstrates that key regulators of some BGCs with putative ecological functionalities may have important epistatic interactions, raising tantalizing questions about the ecological consequences of such interactions. Indeed, the co-option of a trait for a purpose that it did not originally evolve is referred to as exaptation and has unique evolutionary and ecological implications for the process of adaptation (47). Given the mobile nature of BGCs (48), we suggest that such exaptation may be of particular importance to our understanding of secondary metabolism in fungi.

Although *PexanC* positively regulates citrinin production in *P. expansum*, it is not essential for the production of citrinin, as determined by examination of the *ΔPexanC* strain (TWW17.1) in PDA medium (Fig. 1d). In contrast, deletion of the *cit* BGC TF, CtnA, is essential for *cit* gene expression and metabolite production (Fig. 2d and e). Our work demonstrated that *P. expansum* CtnA is the pathway-specific regulator for the *cit* BGC and is required for citrinin synthesis. This is the first report of TF regulation of the *cit* BGC in *P. expansum* or any *Penicillium* spp. While the function of CtnA has been explored in *Monascus* spp., it has not been found to be consistent. One study showed that disruption of *ctnA* resulted in decreased expression of the citrinin synthase gene *citS* (also called *pksCT*) and the dehydrogenase gene *ctnC* (also called *orf5*) as well as reduction of citrinin production in *M. purpureus* (32), whereas another group found *citS* transcription to be decoupled from *ctnA* expression in the same species (49). A third study of another *M. purpureus* isolate found that replacing *ctnA* with a *pks* gene resulted in a 42% reduction but not elimination of citrinin synthesis (50). Five strains of *M. ruber* which did not produce citrinin were found to lack the *ctnA* gene while retaining *citS* (51). Together, these studies suggest some variation of CtnA function in *Monascus* spp. Although a complete understanding of *cit* BGC regulation in fungi remains unclear, our work establishes PeXanC as a new positive regulator for *cit* BGC in *P. expansum*. A future direction is to determine if XanC homologs in other species also regulate expression of *cit*-like BGCs (Fig. 5).

This is also the first report that the growth of *P. expansum* could be affected by high production of citrinin. Figure 2b and c shows that deletion of *ctnA* results in expanded colony growth on PDA medium, the medium that results in the highest levels of citrinin (Fig. 2e). Citrinin was originally characterized as an antibiotic with bacteriostatic, antifungal, and antiprotozoal properties (52, 53). It was considered to be an agent of chemical offense, and this was supported by observations that the *citS* and/or *pksCT* mutants were reduced in the ability to colonize apples and that this capacity was restored by adding external citrinin (54). Although it is still not clear in *P. expansum*, there are data showing that SM-producing fungi have a self-protection system(s) to avoid self-harm from endogenous SMs (55). Specifically addressing citrinin production, one study showed no significant difference in a comparison of the growth of a wild-type (WT) strain with that of citrinin-deficient mutants in *P. expansum* PEX1 (56), while another study mentioned that the inconsistent cellular growth of a *ctnA* complemented *Monascus purpureus* strain could result by the uncontrolled production of citrinin to levels harmful to cellular growth (32). It is not possible to directly compare our results to the above-described *P. expansum* study, as the concentration of citrinin was not reported and the lack of difference in growth in PEX1 mutants could possibly be attributed to a generally low level of citrinin synthesis in that study.

How XanC TFs function remains unknown. BLAST analysis annotates all XanC proteins as bZIP proteins. Although we did not identify a canonical leucine zipper in either AfXanC or PeXanC (Fig. 3a), there is enough flexibility in this region that XanC proteins are not precluded as bZIP proteins (57). bZIP proteins function as dimers, as either homo- or heterodimers. If XanC proteins are bZIP TFs, it is possible that AfXanC and PeXanC could have different association partners, which would almost certainly affect their binding targets (58). We note that several bZIP proteins have been associated with changes in secondary metabolite production in fungi. A salient case is that of RsmA, a bZIP protein that regulates the sterigmatocystin BGC by directly binding to the promoter of AflR, the sterigmatocystin in-cluster TF (59), thus showing a parallel *trans*-regulation scheme similar to PeXanC regulation of CtnA. RsmA itself is not located in a BGC; however, this is true for most bZIP TFs that impact BGC expression and product formation (60). Along with XanC, another example of an in-cluster bZIP TF is the bZIP (termed OTAR1 or AcOTAbZIP, depending on the species) that regulates the ochratoxin BGC in Aspergillus spp. (61, 62).

An ability to predict regulatory changes in BGC expression would greatly aid efforts in natural product mining of fungal metabolites and would guide research or surveillance for toxigenic isolates of agricultural or medicinal fungal pathogens. Although we do not know when the regulatory deviation of AfXanC and PeXanC occurred, we can speculate on environmental forces that might have impacted the evolutionary processes leading to this divergence. For instance, the A. fumigatus *xan* BGC is transcriptionally responsive to external copper levels and is regulated by two copper homeostasis TFs (AceA and MacA). Furthermore, its products are increased during copper starvation (20). Most recently, the *xan* BGC isocyanide moieties have been demonstrated to be the first eukaryotic copper binding natural products with evidence that they participate in copper homeostasis for A. fumigatus (26, 63). Interestingly, citrinin also has metal complexing properties and could react with copper(II) to form 1:1 and 1:2 chelates (64). We speculate that XanC evolution might be linked to the regulation of copper-chelating natural products and possible copper homeostasis in the *Eurotiales* and note the involvement of the bZIP protein HapX in iron homeostasis in Aspergillus spp. (65).

## MATERIALS AND METHODS

### Growth and culture conditions of strains.

All fungal strains were cultivated in glucose minimal medium (GMM) (66) agar at 25°C for 4 days to collect fresh spores. Spores were harvested with 0.1% Tween 80 and adjusted to desired concentrations using a hemocytometer. For isolation of genomic DNA (gDNA) for PCR and Southern blotting, 10 ml of liquid minimal medium with 0.5% yeast extract was inoculated with 1.0 × 10^7^ spores and grown for 2 days at 25°C. For secondary metabolite analysis, strains were point inoculated in solid GMM and PDA plates with 10 μl of spore suspension containing 5.0 × 10^5^ spores and grown for 14 days at 25°C. All strains were maintained as glycerol stocks at −80°C.

### Mutant construction.

A yeast recombination system (67) was used to generate plasmids containing the DNA constructs for transformation as described previously (68). All the plasmids constructed are listed in Table S2 in the supplemental material. The linearized plasmid pE-YA (69) by NotI and AscI was used as the empty vector to contain a Hyg^r^, *pyrG*, or *argB* selective marker and 1 kb sequence flanking the target gene for homologous recombination. Flanking sequences were amplified with 20-bp overlaps using primers designed with Primer Premier 6 (Premier Biosoft). In this study, we used the recyclable β-Rec/*six* site-specific recombination system (70) for all hygromycin resistance cassettes (*β-rec*/*six*::Hyg^r^). Selective markers were amplified from either pSK529 (*β-rec*/*six*::Hyg^r^) (70, 71) or A. fumigatus (*pyrG* and *argB*). Deletion mutants were constructed by whole-gene deletion, and overexpression mutants were created by inserting a constitutively active A. nidulans *gpdA* promoter upstream of the ATG translation start site of a gene. The constructs were then transformed into P. expansum and A. fumigatus as described previously (72).

10.1128/mBio.01399-21.2 TABLE S2 Plasmids and strains used in this study. Download Table S2, PDF file, 0.6 MB. Copyright © 2021 Wang et al. 2021 Wang et al. https://creativecommons.org/licenses/by/4.0/ This content is distributed under the terms of the Creative Commons Attribution 4.0 International license.

### (i) *Penicillium expansum* mutants.

*P. expansum Δku70* strain TJT14.1 (27) was used as the initial parental strain for construction of subsequent strains. A *pyrG* auxotroph, TDL9.1, was constructed by replacing *pyrG* with the *β-rec*/*six*::Hyg^r^ selective marker in TJT14.1. Then, TDL9.1 was used to construct the *pyrG* complement strain TDL12.1, the *PexanC* overexpression strain TWW4.1/4.2/4.3, the *PexanC* deletion strain TWW17.1, and the OE::*AfXanC*-*Pe* strain TWW5.1. To determine if PeXanC induced *cit* gene expression *via* CtnA, first the Hyg^r^ selective marker was excised in TDL12.1 and TWW4.1 by growing them on minimal medium amended with 2% xylose (27) to produce strains TWW13.1 and TWW14.1 respectively. The *ctnA* deletion cassette was then transformed into TWW13.1 and TWW14.1 to create the *ΔctnA* strain TWW19.1 and the *ΔctnA* OE::*PexanC* double mutant TWW18.1. To check the predicted recognition motif of PeXanC, the motif (5′-TGGNTGNG-3′) deletion construct was transformed into TWW14.1 to build TWW27.1. The motif deletion in the *ctnA* promoter was made by using designed primers. Two pairs of primers (RH1_motif_F/R, RH2_motif_F/R) were used to amplify the *ctn*A promoter with motif deletion in inner primers. The motif-deleted promoter region was then transformed into TWW14.1 with the Hyg^r^ selective marker and 1 kb left flanking sequence. TWW27.1 was compared with control strain TDL12.1 and OE::*PeXanC* strain TWW4.1.

### (ii) Aspergillus fumigatus mutants.

A. fumigatus OE::*xanC argB* auxotrophic strain TNLR11.3 (26) was used to build binding site mutant TWW31.1 and binding site control TWW32.1. The *AfxanB* promoter flanking sequence containing mutagenesis of 5′-AGTCAGCA-3′ to 5′-AGTCTTTA-3′ was made by using designed primers. Two binding sites in the *AfxanB* promoter split it into three fragments. Three pairs of primers (RH1_BS_F/RH1_BSm_R, RH2_BSm_F/R, RH3_BSm_F/RH3_BS_R) were used to amplify the *AfxanB* promoter and containing binding site mutagenesis in all inner primers. The construct containing the mutational *AfxanB* promoter, the *argB* selective marker, and 1 kb left flanking sequence was then transformed into TNL11.3 to build TWW31.1 (BSm OE::*AfxanC*). To eliminate the effect of the selective marker, a control construct with the native *AfxanB* promoter, the *argB* selective marker, and 1 kb left flanking sequence was transformed into TNL11.3 to build TWW32.1 (BSc OE::*AfxanC*). Both strains were then compared with A. fumigatus WT TFYL81.5, OE::*AfxanC* strain TNLR1.2, and *ΔAfxanC* strain TNLR9.1 (26).

All transformants were screened by PCR using the marker forward primer and the 3′ flanking reverse primer. Positive mutants were further confirmed by Southern blot analysis using ^32^P-labeled 1-kb flanking regions described previously (27) to confirm single integrations (Fig. S2). All strains used in this study are listed in Table S2, and all primers used to create and confirm the mutant strains are listed in Table S3.

10.1128/mBio.01399-21.3 TABLE S3 Primers used in this study. Download Table S3, PDF file, 0.4 MB. Copyright © 2021 Wang et al. 2021 Wang et al. https://creativecommons.org/licenses/by/4.0/ This content is distributed under the terms of the Creative Commons Attribution 4.0 International license.

### RNA extraction and semi-qPCR/qPCR analysis.

GMM agar plates overlaid with sterilized cellophane discs were point inoculated with 5 × 10^5^ spores and allowed to incubate at 25°C for 3 days. Total RNA was then extracted using TRIzol (Invitrogen). A 10-μg volume of total RNA was treated with DNase I (New England Biolabs), and cDNA was synthesized using an iScript cDNA synthesis kit (Bio-Rad) with 500 ng DNase I-treated RNA.

Semiquantitative PCR (semi-qPCR) was performed with a template of 1 μl diluted cDNA (diluted 5 times) and 0.25 μl *Taq* polymerase (Promega) in a 12.5-μl reaction mixture. The PCR was carried out as follows: 5 min at 95°C, 25 cycles of 95°C for 30 s, 60°C for 30 s, and 72°C for 30 s, and a hold at 4°C.

Quantitative PCR (qPCR) was performed with a template of 1 μl diluted cDNA (diluted 10 times) and 5 μl iQ SYBR green supermix (Bio-Rad) in a 20-μl reaction mixture. The qPCR was carried out in QuantStudio 7 flex real-time PCR systems (Thermo Fisher) as follows: 3 min at 95°C, 40 cycles of 95°C for 10 s and 60°C for 30 s, and a melt curve with 55 to 95°C, at an 0.5°C increment and 2 s/step. The primers used for each of the indicated genes are listed in Table S3.

### Metabolite extraction, liquid chromatography-mass spectrometry (LC-MS) analysis, and quantitative analysis.

Six plugs (15 mm in diameter) from GMM and PDA plates were cut into small pieces using a spatula and placed in a 20-ml glass vial. Ten milliliters of ethyl acetate was added to each vial, and samples were sonicated for 180 min. Ten milliliters of water was then added to each sample, and the vials were shaken for 5 s. Samples were left at room temperature for 10 min to allow for separation of the two layers. The ethyl acetate layer was then moved to a new glass vial for evaporation to dryness.

Crude extracts were resuspended in 0.5 ml of acetonitrile with 20% water and filtered using 0.2-μm Target2 polytetrafluoroethylene (PTFE) syringe filters (Thermo Fisher). Ten-microliter volumes of samples were subjected to ultrahigh-performance liquid chromatography coupled with high-resolution mass spectrometry (UPLC-HRMS), which was performed on a Thermo Scientific-Vanquish UPLC system connected to a Thermo Scientific Q-Exactive Orbitrap mass spectrometer in ES^+^ and ES^−^ modes between 200 *m/z* and 1,000 *m/z* to identify metabolites. A Zorbax Eclipse XDB-C_18_ column (2.1 by 150 mm, 1.8-μm particle size) was used with 0.05% formic acid in acetonitrile (organic phase) and 0.05% formic acid in water (aqueous phase) as solvents at a flow rate of 0.2 ml/min. A solvent gradient scheme was used, starting at 20% organic for 2 min, followed by a linear increase to 98% organic over 13 min, holding at 98% for 5 min, decreasing back to 20% organic for 1 min, and holding at 20% organic for the final 4 min, for a total of 25 min. Data acquisition and procession for the UPLC-MS were controlled by using Thermo Scientific Xcalibur software. Files were converted to the .mzXML format using MassMatrix MS data file conversion and analyzed by MAVEN (73) and XCMS (74).

To quantify the production of citrinin in different mutants, a standard curve was built using a series of diluted (from 0.001 mg/ml to 1 mg/ml) citrinin standards (Cayman Chemical) by high-performance liquid chromatography (HPLC; Gilson) with a 10-μl injection. A Waters XBridge C_18_ column (4.6 by 100 mm, 3.5-μm particle size) was used with 0.1% formic acid in acetonitrile (organic phase) and 0.1% formic acid in water (aqueous phase) as solvents at a flow rate of 0.8 ml/min. A solvent gradient scheme was used, starting at 20% organic for 2 min, followed by a linear increase to 95% organic over 18 min, holding at 95% for 1 min, increasing to 100% in 0.1 min and holding at 100% organic for 1.9 min, decreasing back to 20% organic for 0.1 min, and holding at 20% organic for the final 1.9 min, for a total of 25 min. The standard curve was built linking the concentration and the peak area at 330 nm and was formulated as *y* = 23,110*x* with an *r*^2^ of 0.9833. The concentration of citrinin in different strains was then calculated based on the standard curve (Data Set S1).

To compare the production of xanthocillin derivatives in the A. fumigatus control, *ΔAfxanC*, and OE::*AfxanC* strains and the BSm OE::*AfxanC* and BSc OE::*AfxanC* double mutants, the peak intensity of xanthocillin derivative characterized ions (20) was collected using UPLC-HRMS (Data Set S2). As standards were lacking for these compounds, the relative production in different strains is presented (Fig. 3g).

### Statistical analysis.

For all experiments, values are given as the mean ± standard deviation (SD) of four independent replicates. The statistical analysis of the data was performed using one-way analysis of variance (ANOVA). If one-way ANOVA reported a *P* value of <0.05, further analysis was performed using Tukey’s single-step multiple comparison test to determine the significant difference between the strains. Analyses were done using GraphPad Prism version 8 for Windows (GraphPad Software).

### Prediction of DNA binding sites.

Promoter sequences are defined as 1,000 bp located directly upstream of the start codon or as the nucleotide sequence between two genes when less than 1,000 bp. Fifty promoter sequences of *xan* genes were collected from 16 Aspergillus sp. and *Penicillium* sp. strains for searching the binding site of AfXanC. Nine *ctnA* promoters and the *PexanG* promoter were collected for searching the binding site of PeXanC. The sequence files were then analyzed by MEME (37) (https://meme-suite.org).

### Putative *xan* and *cit* BGC analysis.

To identify species with putatively intact xanthocillin and citrinin gene clusters, we used existing protein sequences from A. fumigatus and *P. expansum* (respectively) to query the NCBI genome database *Eurotiales* data set (downloaded on 20 April 2020) using BLASTP and tBLASTN implemented in the BLAST+ suite v2.8. (75). The resulting hits were filtered to include the species with the most BLAST hits within 25 kb of either side of the BGC’s backbone gene (*xanB* or *citS*). Additionally, we selected a subset of species that had previously been reported to produce citrinin or xanthocillin (22, 40, 76–78). When multiple genes in a single genome shared similar levels of protein identity with the backbone gene, the gene surrounded by the highest number of clustered hits was selected. In all selected species, we looked for XanC protein sequences by performing a reciprocal best hit BLAST search between target genomes and the A. fumigatus CEA10 genome (GCA_000150145.1) by using a method described previously (79). XanC sequences were aligned using Clustal Omega (80) and MEGA v10.0. (81). The resulting alignment was used to construct a maximum likelihood phylogeny in the same program.

### Data availability.

The nucleotide sequence of *PexanC* is available in the Third Party Annotation (TPA) section of the DDB/ENA/GenBank database under accession number TPA BK013036.
