# Supplementary figures and images for: Transcription Factor Repurposing Offers Insights into Evolution of Biosynthetic Gene Cluster Regulation
Source: mBio. 2021 Jul 20;12(4):e01399-21. doi: 10.1128/mBio.01399-21 (PMC8406171; doi:10.1128/mBio.01399-21)

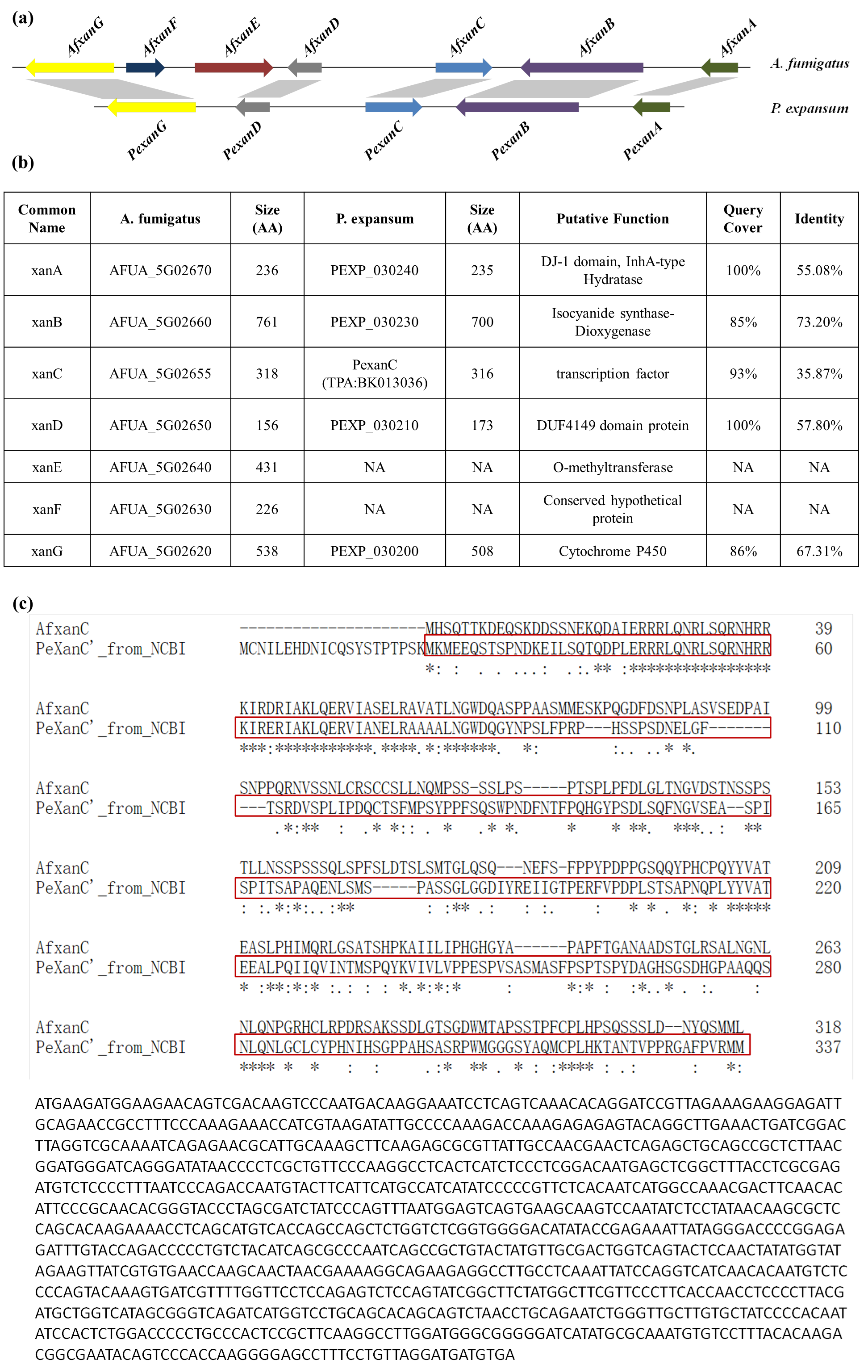

Supplement: FIG S1 [file mbio.01399-21-sf001.tif]

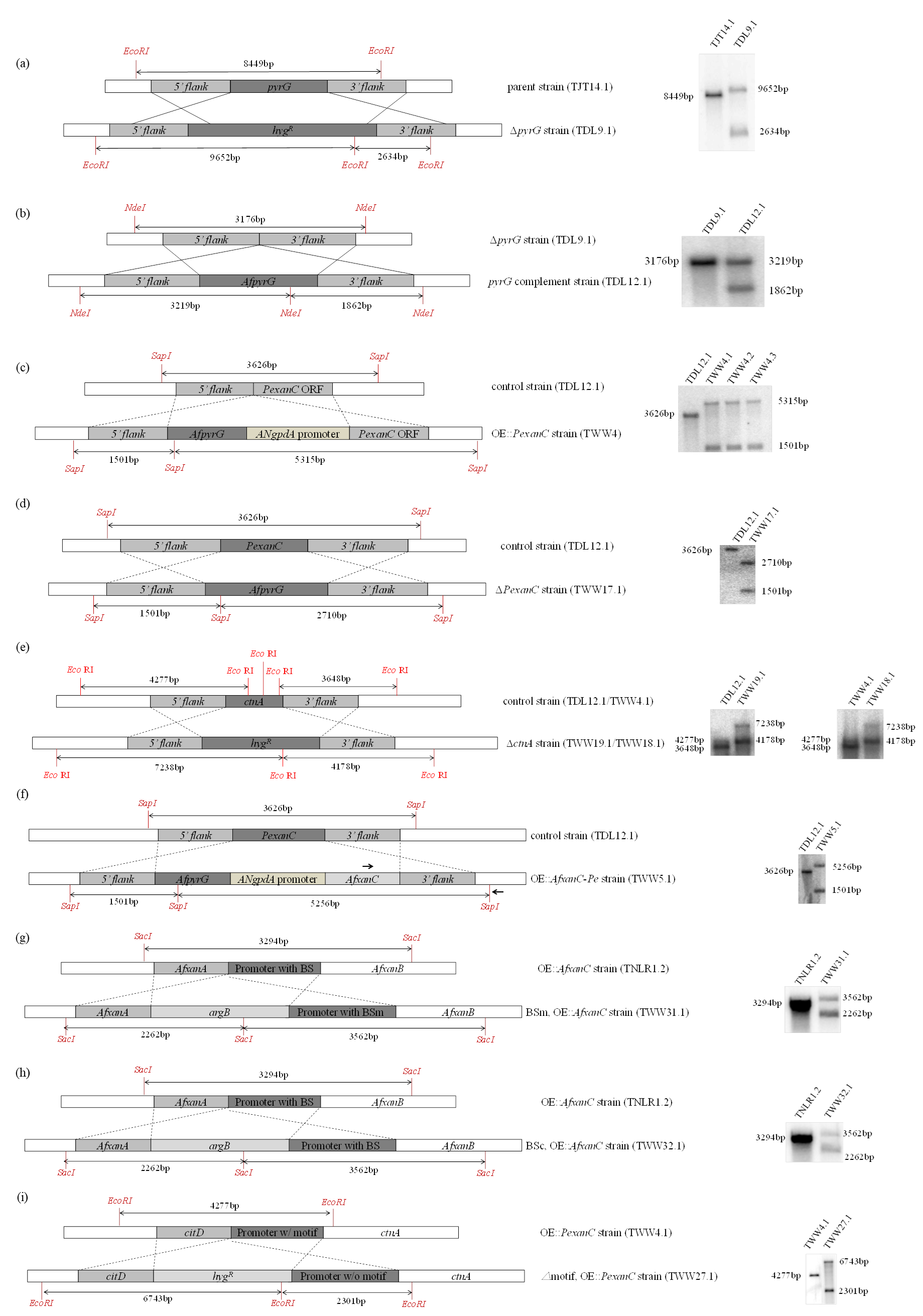

Supplement: FIG S2 [file mbio.01399-21-sf002.tif]

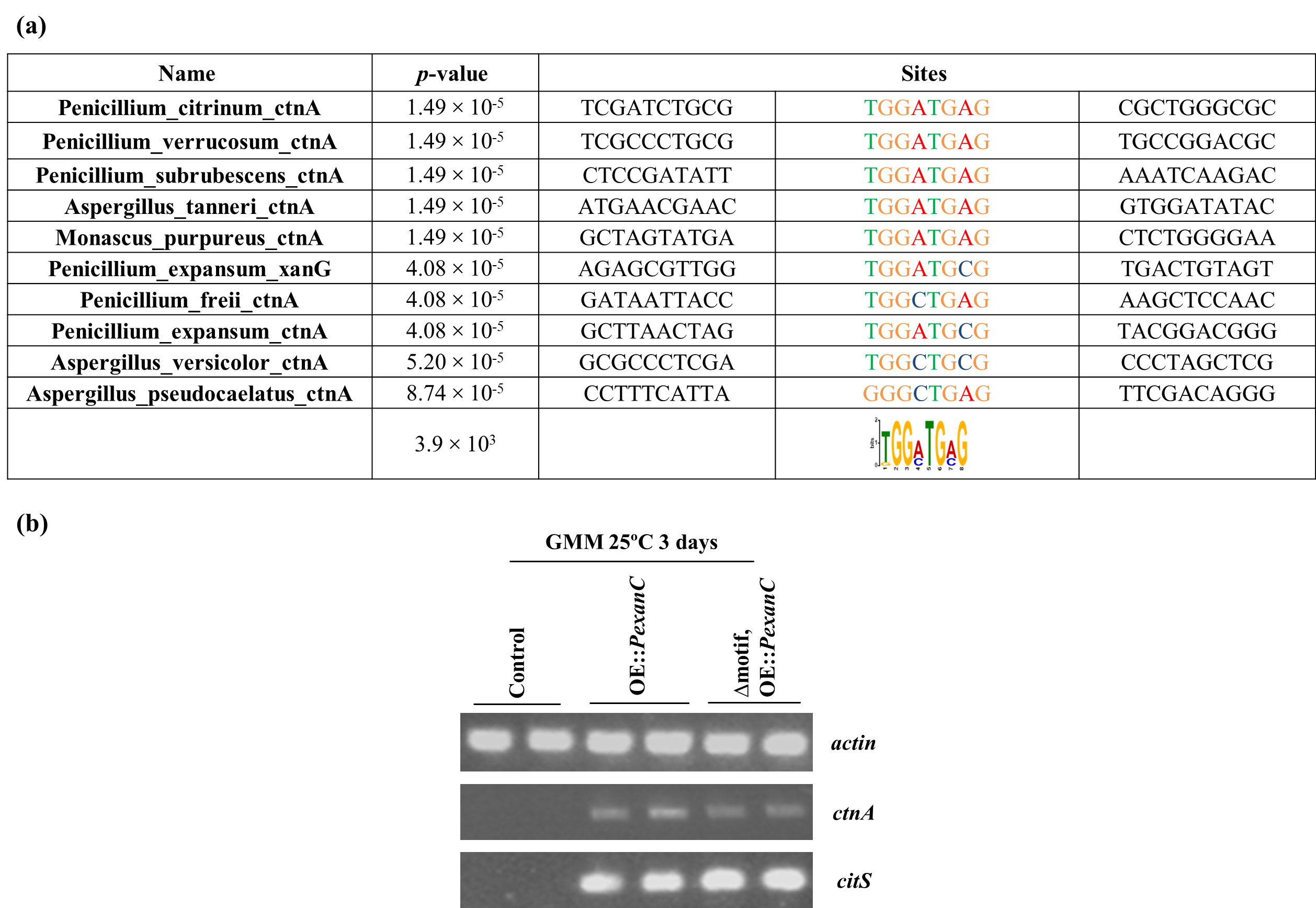

Supplement: FIG S4 [file mbio.01399-21-sf004.tif]

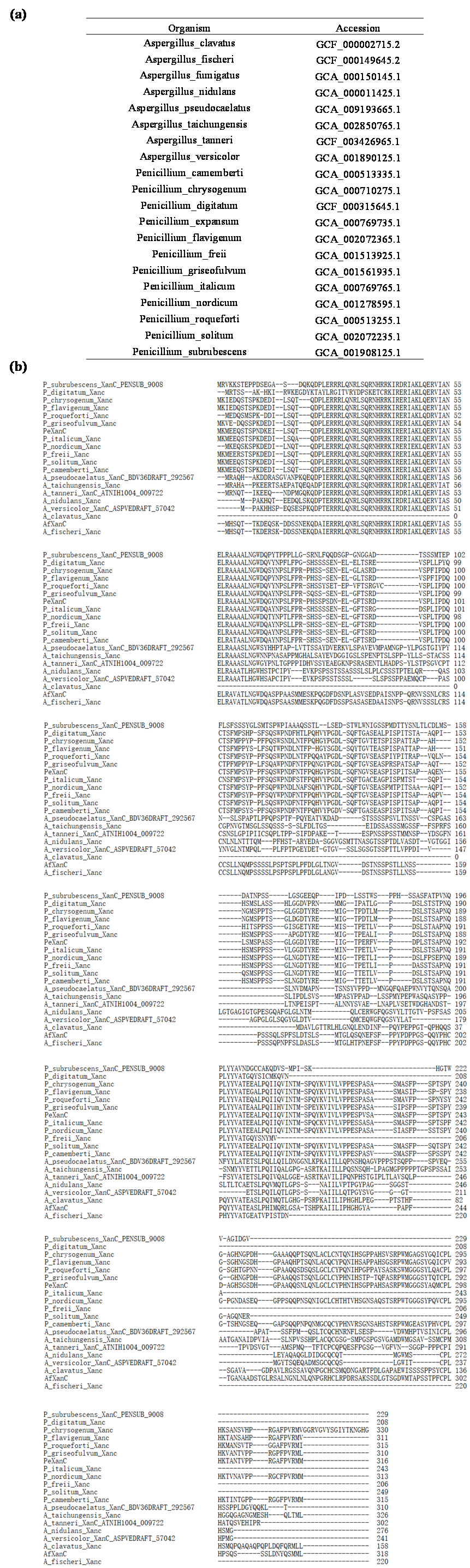

Supplement: FIG S3 [file mbio.01399-21-sf003.tif]
