# Supplementary material for: Transcription Factor Repurposing Offers Insights into Evolution of Biosynthetic Gene Cluster Regulation
Source: mBio. 2021 Jul 20;12(4):e01399-21. doi: 10.1128/mBio.01399-21 (PMC8406171; doi:10.1128/mBio.01399-21)
Supplement: TABLE S1 [file mbio.01399-21-st001.pdf]

Table S1 Search of AfXanC binding site 5’-AGTCAGCA-3’ in the promoters of *xan* genes in the *Eurotiales* spp. and specific in *Pexan* genes and *cit* genes in *P. expansum*.

AfXanC binding site 5’-AGTCAGCA-3’ in the promoters of *xan* genes in the *Eurotiales* spp.			
**Name**	**Sites**		
AfxanA	GTGCAGATGT	AGTCAGCA	ATGTCAGCTT
AfxanB	TGGACGGCCC	AGTCAGCA	GACATGCTGC
AfxanB	GAATTTTGGC	AGTCAGCA	TGATCCACAG
AfxanG	ATGCATCCCA	AGTCAGCA	AATCCGAGCT
AfxanE	GGCCAGCTAC	AGTCAGCA	TTGGTCGTAA
xanA_A_fischeri	GTGCAGATGT	AGTCAGCA	GTGTCAGCTT
xanB_A_fischeri	AAGACGACCC	AGTCAGCA	GATATGCTGC
xanB_A_fischeri	GGATTTTGGC	AGTCAGCA	TCATCTATAG
xanE_A_fischeri	GACCAGCTAC	AGTCAGCA	TCGGTCGTAA
xanG_A_fischeri	ATGCATCCCA	AGTCAGCA	AATCCGAGCT
xanA_A_clavatus	GTGCAGGCGG	AGTCAGCA	GTGTCTGTCA
xanE_A_clavatus	GCCACCTTAC	AGTCAGCA	TAGTTCGTGT
xanG_A_clavatus	ATGCATTCCA	AGTCAGCA	AACACGGGCT
PexanA	TTTTGGCCGG	AGTCAGCA	GAGTCAGAAA
xanA_P_flavigenum	TTATTTTCGG	AGTCAGCA	GAGTCAGAAA
xanA_P_nordicum	TTTACGCCGG	AGTCAGCA	GAGTCAGAAA
xanA_P_griseofulvum	TTTACGCCGG	AGTCAGCA	GAGTCAGAAA
xanA_P_camemberti	TTTACGCCGG	AGTCAGCA	GAGTCAGAAA
xanA_P_solitum	TTTACGCCGG	AGTCAGCA	GAGTCAGAAA
xanA_P_ferii	TTTACGCCGG	AGTCAGCA	GAGTCAGAAA
xanA_P_digitatum	GTTTTGCTGG	AGTCAGCA	GAGTCAGAAA
xanA_P_roqueforti	TTGTTTTCGG	AGTCAGCA	GAGTCAGAAA
xanA_P_chrysogenum	TTATTTTCGG	AGTCAGCA	GAGTCAGAAA
xanG_P_griseofulvum	TTGTATAACA	AGTCAGCA	GGGTTGGAGC
AfXanC DNA binding site in *Pexan* promoters and *cit* promoters			
PexanA	TTTTGGCCGG	AGTCAGCA	GAGTCAGAAA
PexanB		ND	
PexanC		ND	
PexanG		ND	
all cit genes		ND	
