# Supplementary material for: Transcription Factor Repurposing Offers Insights into Evolution of Biosynthetic Gene Cluster Regulation
Source: mBio. 2021 Jul 20;12(4):e01399-21. doi: 10.1128/mBio.01399-21 (PMC8406171; doi:10.1128/mBio.01399-21)
Supplement: TABLE S2 [file mbio.01399-21-st002.pdf]

Table S2 Plasmids and strains used in this study

**Plasmids**	**Genotype**	**Vector**	**Source**
**pSK529**	^{P}*xylP::β-rec::trpC*^{t}*::hyg*^{R}*::*^{P}*gpdA* (*β-rec/six::hyg*^{R})		**(1)**
**pE-YA**	***Kan***^{R}***, URA3, ori, 2μ***		**(2)**
**pWW2**	***AfpyrG::ANgpdAp::PexanC, Kan***^{R}***, URA3, ori, 2μ***	**pE-YA**	**This study**
**pWW3**	***△PexanC::AfpyrG, Kan***^{R}***, URA3, ori, 2μ***	**pE-YA**	**This study**
**pWW4**	*△ctnA::β-rec/six::hyg*^{R}, ***Kan***^{R}***, URA3, ori, 2μ***	**pE-YA**	**This study**
**pWW9**	*△motif*[a]*::β-rec/six::hyg*^{R}, ***Kan***^{R}***, URA3, ori, 2μ***	**pE-YA**	**This study**
**pWW13**	***BSm***[b]***::argB, Kan***^{R}***, URA3, ori, 2μ***	**pE-YA**	**This study**
**pWW14**	***BSc***[c]***::argB, Kan***^{R}***, URA3, ori, 2μ***	**pE-YA**	**This study**
**pWW21**	***AfpyrG::ANgpdAp::AfxanC, Kan***^{R}***, URA3, ori, 2μ***	**pE-YA**	**This study**

**Strains**	**Genotype**	**Parent strain**	**Reference**
**TJT14.1**	***Δku70::hyg***^{R}***, hyg***^{R}***-***	**Pe-21**	**(3)**
**TDL9.1**	***ΔpyrG::hyg***^{R}***, Δku70::hyg***^{R}***, hyg***^{R}***-***	**TJT14.1**	**This study**
**TDL12.1**	***Δku70::AfpyrG+, ΔpyrG::hyg***^{R}***, Δku70::hyg***^{R}***, hyg***^{R}***-***	**TDL9.1**	**This study**
**TWW4.1/4.2/4.3**	***AfpyrG::ANgpdAp::PexanC, ΔpyrG::hyg***^{R}***, Δku70::hyg***^{R}***, hyg***^{R}***-***	**TDL9.1**	**This study**

**TWW5.1**	***AfpyrG::ANgpdAp::AfxanC, ΔpyrG::hyg***^{R}***, Δku70::hyg***^{R}***, hyg***^{R}***-***	**TDL9.1**	**This study**
**TWW13.1***	***Δku70::AfpyrG+, ΔpyrG::hyg***^{R}***, hyg***^{R}***-, Δku70::hyg***^{R}***, hyg***^{R}***-***	**TDL12.1**	**This study**
**TWW14.1***	***AfpyrG::ANgpdAp::PexanC, ΔpyrG::hyg***^{R}***, hyg***^{R}***-, Δku70::hyg***^{R}***, hyg***^{R}***-***	**TWW4.1**	**This study**
**TWW17.1**	***ΔPexanC::AfpyrG, ΔpyrG::hyg***^{R}***, Δku70::hyg***^{R}***, hyg***^{R}***-***	**TDL9.1**	**This study**
**TWW18.1***	***ΔctnA::hyg***^{R}***, AfpyrG::ANgpdAp::PexanC, ΔpyrG::hyg***^{R}***, hyg***^{R}***-, Δku70::hyg***^{R}***, hyg***^{R}***-***	**TWW14.1**	**This study**
**TWW19.1***	***ΔctnA::hyg***^{R}***, Δku70::AfpyrG+, ΔpyrG::hyg***^{R}***, hyg***^{R}***-, Δku70::hyg***^{R}***, hyg***^{R}***-***	**TWW13.1**	**This study**
**TFYL81.5**	***pyrG-, argB-, fumiargB, fumipyrG, ΔakuA::mluc***		**(4)**
**TNLR1.2**	***pyrG-, argB-, fumiargB, parapyrG::ANgpdAp::AFUA_5G02655, ΔakuA::mluc***		**(5)**
**TNLR9.1**	***pyrG-, argB-, fumiargB, ΔAFUA_5G02655:: parapyrG, ΔakuA::mluc***		**(5)**
**TNLR11.3**	***pyrG-, argB-, parapyrG::ANgpdAp::AFUA_5G02655, ΔakuA::mluc***		**(6)**
**TWW31.1**	***BSm::argB, pyrG-, argB-,***	**TNLR11.3**	**This study**

	parapyrG::ANgpdAp::AFUA_5G02655, ΔakuA::mluc		
**TWW32.1**	***BSc::argB, pyrG-, argB-, parapyrG::ANgpdAp::AFUA_5G02655, ΔakuA::mluc***	**TNLR11.3**	**This study**
**TWW29.1**	***△motif::hyg^R, AfpyrG::ANgpdAp::PexanC, ΔpyrG::hyg^R, hyg^R-, Δku70::hyg^R, hyg^R-***	**TWW14.1**	**This study**

[a], motif is the motif 5’-TGGNTGNG-3’ in *ctnA* promoter.

[b], BSm is short for binding site mutant.

[c], BSc is short for binding site control.

[*], strains contain two ‘*hyg^R*-’ in their genotype. We recycled hygromycin selective marker twice usingβ-Rec/six site-specific recombination system.

AfpyrG: *pyrG* from *A. fumigatus*; *ANgpdAp*: *gpdA* promoter from *A. nidulans*; *fumiargB*: *argB* from *A. fumigatus*; *parapyrG*: *pyrG* from *A. parasiticus*.

1. Hartmann T, Dumig M, Jaber BM, Szewczyk E, Olbermann P, Morschhauser J, Krappmann S. 2010. Validation of a self-excising marker in the human pathogen *Aspergillus fumigatus* by employing the beta-Rec/*six* site-specific recombination system. Appl Environ Microb 76:6313-6317.
2. Pahirulzaman KA, Williams K, Lazarus CM. 2012. A toolkit for heterologous expression of metabolic pathways in *Aspergillus oryzae*. Methods Enzymol 517:241-60.
3. Tannous J, Kumar D, Sela N, Sionov E, Prusky D, Keller NP. 2018. Fungal attack and

host defence pathways unveiled in near-avirulent interactions of *Penicillium expansum creA* mutants on apples. Mol Plant Pathol 19:2635-2650.

4. Throckmorton K, Lim FY, Kontoyiannis DP, Zheng W, Keller NP. 2016. Redundant synthesis of a conidial polyketide by two distinct secondary metabolite clusters in *Aspergillus fumigatus*. Environ Microbiol 18:246-59.

5. Lim FY, Won TH, Raffa N, Baccile JA, Wisecaver J, Rokas A, Schroeder FC, Keller NP. 2018. Fungal Isocyanide Synthases and Xanthocillin Biosynthesis in *Aspergillus fumigatus*. mBio 9:E00785-18.

6. Raffa N, Won TH, Sukowaty A, Candor K, Cui C, Halder S, Dai M, Landero-Figueroa JA, Schroeder FC, Keller NP. 2021. Dual-purpose isocyanides produced by *Aspergillus fumigatus* contribute to cellular copper sufficiency and exhibit antimicrobial activity. Proc Natl Acad Sci U S A 118:E2015224118.
