# Supplementary material for: Transcription Factor Repurposing Offers Insights into Evolution of Biosynthetic Gene Cluster Regulation
Source: mBio. 2021 Jul 20;12(4):e01399-21. doi: 10.1128/mBio.01399-21 (PMC8406171; doi:10.1128/mBio.01399-21)
Supplement: TABLE S3 [file mbio.01399-21-st003.pdf]

Table S3 Primers used in this study.

Primers used for constructs generation and PCR confirmation of mutants

Strain	Product	Primer	Sequence (5'-3')
TDL9.1	LH	LH_dpyrG_F<br>LH_dpyrG_R	AGGGTCATTGGAAACTGCATATTCTGG<br>CTCTATTGACCTATAGGACCTGAGTGATGCTGTGTTTTGATGTCTCCAGAGGATGG
	Hyg^R	HygR _F<br>HygR _R	GCATCACTCAGGTCCTATAGGTCAATA<br>GCACTAGATGGACCATATTATGCTCAAC
	RH	RH_dpyrG_F<br>RH_dpyrG_R	AAGTTGAGCATAATATGGTCCATCTAGTGCAGCGTGCAGTCTACGTTCCACTG<br>GAAGCAGGCGTGTTTAGTATGTTCC
	construct	dpyrG_Fin<br>dpyrG_Rin	CACAAAAGTAGACAAGGTAACCCTTGG<br>TCGGGTCTGCTTTCTTAATATTCTTCC
	confirmation	ScreenPCR_dpyrG_F<br>ScreenPCR_dpyrG_R	TGTTAGTCAAGCTGCGATGAAGTGG<br>TCGGGTCTGCTTTCTTAATATTCTTCC
TDL12.1	LH	LH_CpyrG_F<br>LH_CpyrG_R	AAGTGCCTGAATGAAGACTGGGC<br>CACAGTGGAGGACATACCCGTAATTTTCTGGGTTGTGAAACAGGGAACCTTAGAG
	pyrG	PyrG_F<br>PyrG_R	CAGAAAATTACGGGTATGTCCTCCAC<br>TCATGACTTGCCGCATACTCTGG
	RH	RH_CpyrG_F<br>RH_CpyrG_R	TATATGGCCAGAGTATGCGGCAAGTCATGAACGCTGATTGAAGTTTGCTTGGGATC<br>CTGACAGGAAAGATTCCCATGATGC
	construct	CpyrG_Fin<br>CpyrG_Rin	AATCCTCCATTTGCGCGCTTTCC<br>GCTTCAATATCTGGGATAGAGCTTTGG
	confirmation	ScreenPCR_CpyrG_F<br>ScreenPCR_CpyrG_R	CTACGCTCGCAAATACAAGAACTTCG<br>AGCATAGGTTTGACCCAAAATAGCTGC
TWW4	LH	LH_PexanC_F<br>LH_PexanC_R	GGAGGTGCCTAGTGACAGAT<br>TCTGACTTCAACACAGTGGAGGACATACCCGTAATTTTCTGGGAAAATTACGCCTGATC
	pyrG	PyrG_F<br>PyrG_R	CAGAAAATTACGGGTATGTCCTCCAC<br>TCATGACTTGCCGCATACTCTGG
	gpdAp	ANgpdAp_F<br>ANgpdAp_R	GCTGGGAAGCTTATATGGCCAGAGTATGCGGCAAGTCATGACCTGCGTTGGTTGAATTT<br>AAGCGGGGTAGCTGTTAG
	RH	ORF_PexanC_F<br>ORF_PexanC_R	TTTCCCACTTCATCGCAGCTTGACTAACAGCTACCCCGCTTATGAAGATGGAAGAACAG<br>GGACTGTATTCGCCGTCT
	construct	PexanC_Fin<br>OE PexanC_Rin	ACGAGTGGTTTAGCTCAAACCC<br>GGACTGTATTCGCCGTCT
	confirmation	ScreenPCR_OEPexanC_F<br>ScreenPCR_OEPexanC_R	TTGCGTCAGTCCAACATT<br>ACCAGTGCGTGAACCTAT
TWW17.1	LH	LH_PexanC_F<br>LH_PexanC_R	GGAGGTGCCTAGTGACAGAT<br>TCTGACTTCAACACAGTGGAGGACATACCCGTAATTTTCTGGGAAAATTACGCCTGATC
	pyrG	PyrG_F<br>PyrG_R	CAGAAAATTACGGGTATGTCCTCCAC<br>TCATGACTTGCCGCATACTCTGG
	RH	dPexanC RH_F<br>dPexanC RH_R	TGGGAAGCTTATATGGCCAGAGTATGCGGCAAGTCATGAAAAGAGCGTGATTTCTACTA<br>TTCACTTGTCCTGCCACT
	construct	PexanC_Fin<br>dPexanC_Rin	ACGAGTGGTTTAGCTCAAACCC
	confirmation	screenPCR_dPexanC_F<br>screenPCR_dPexanC_R	CGTTATTGCCAACGAACTCA<br>CCCCTTGGTGGGACTGTATT
TWW18.1/TWW19.1	LH	LH_dctnA_F<br>LH_dctnA_R	CTGTGAAGAAACTGCGAACA<br>ATAAGTATACTCTATTGACCTATAGGACCTGAGTGATGCCTAAACAAGAGGGGAAATGC
	Hyg^R	HygR _F<br>HygR _R	GCATCACTCAGGTCCTATAGGTCAATA<br>GCACTAGATGGACCATATTATGCTCAAC
	RH	RH_dctnA_F<br>RH_dctnA_R	AGGTCATTTAAGTTGAGCATAATATGGTCCATCTAGTGCTTATTGGGAAAGGCTGAAAC<br>CAAGCAACCACTGCACTAGG
	construct	dctnA_Fin<br>dctnA_Rin	CGTCGGTCCTGTAGTCTCTC<br>ATGCCCGTACCAACCAAATC
	confirmation	screenPCR_dctnA_F<br>screenPCR_dctnA_R	TTTTGCTTGCTTGACGATCT<br>CTTCACCAGCGTCGTTATTT
TWW5.1	LH	LH_PexanC_F<br>LH_PexanC_R	GGAGGTGCCTAGTGACAGAT<br>TCTGACTTCAACACAGTGGAGGACATACCCGTAATTTTCTGGGAAAATTACGCCTGATC
	pyrG	PyrG_F<br>PyrG_R	CAGAAAATTACGGGTATGTCCTCCAC<br>TCATGACTTGCCGCATACTCTGG
	gpdAp	ANgpdAp_F<br>ANgpdAp_R	GCTGGGAAGCTTATATGGCCAGAGTATGCGGCAAGTCATGACCTGCGTTGGTTGAATTT<br>AAGCGGGGTAGCTGTTAG
	AfXanC ORF	AfxanCORF_F<br>AfxanCORF_R	TTTCCCACTTCATCGCAGCTTGACTAACAGCTACCCCGCTTATGCATTCACAGACTACA<br>GAGTAAAGATTGTTCTCTAGATAGTAGAAATCACGCTCTTTTCAAAGCATCATGGACTG
	RH	RH PexanC_F<br>RH PexanC_R	AAAGAGCGTGATTTCTACTA<br>TTCACTTGTCCTGCCACT
	construct	OE AfxanCPe_Fin<br>OE AfxanCPe_Rin	ACGAGTGGTTTAGCTCAAACCC<br>TTCACTTGTCCTGCCACT
	confirmation	ScreenPCR_OEAfxanCPe_F<br>ScreenPCR_OEAfxanCPe_R	CTTTGAATGGATGGGACC<br>CTCTTCTGTGGCTGTCGG

TWW13.1/14.1	confirmation	HygR_recycle_F<br>HygR_recycle_R	CTTCGTTCACGGCTGTATTAC<br>TATGCTTAGTGGCTTGCTTG
TWW27.1	LH	LH_motif_F<br>LH_motif_R	TTTTATAATGCCAACTTTGTACAAAAAAGCAGGCTGGAAATATCTACTCTAGCGTGATC<br>ATACTCTATTGACCTATAGGACCTGAGTGATGCCTACTTGCATACATCCACTGCGCCAC
	Hyg^R	HygR _F<br>HygR _R	GCATCACTCAGGTCCTATAGGTCAATA<br>GCACTAGATGGACCATATTATGCTCAAC
	RH-1	RH1_motif_F<br>RH1_motif_R	GTCATTTAAGTTGAGCATAATATGGTCCATCTAGTGCCAATCTCTGATAGGTAATTGGA<br>CGTCCGTACTAGTTAAGCATCGCGA
	RH-2	RH2_motif_F<br>RH2_motif_R	TCGCGATGCTTAACTAGTACGGACG<br>CTTATAATGCCAACTTTGTACAAGAAAGCTGGGTGATGCTGGGTCGGTAGACGATTTTC
	construct	motif_Fin<br>motif_Rin	AGTGTGCGTGGGCATCTTACCGTAC<br>ATGCTGGGTCGGTAGACGATTTTCG
	confirmation	ScreenPCR_motif_F<br>ScreenPCR_motif_R	ACTTCGGGGCAGTCCTC<br>CGTCCGTACTAGTTAAGCATCGCGA
TWW31.1	LH	LH_BS_F<br>LH_BS_R	TTTATAATGCCAACTTTGTACAAAAAAGCAGGCTCCGCGGCTATCGCAATTCTGCCTAG<br>TTAATTCGACCACCGAGGATGGCGTGCGAACCAGTTACTCAGAAAGCTGCCCTTTCCAG
	argB	argB_F<br>argB_R	CTGGTTCGCACGCCATCCTC<br>AGCTTGAAGTATTATGGGATG
	RH-1	RH1_BS_F<br>RH1_BSm_R	CACCTACAGAGTACATCATCCCATAATACTTCAAGCTGGGATCTCCCAAAGAGGCGATC<br>ACGGCCCAGTCTTTAGACATG
	RH-2	RH2_BSm_F<br>RH2_BSm_R	CATGTCTAAAGACTGGGCCGTC<br>GAATTTTGGCAGTCTTTATGATCCAC
	RH-3	RH3_BSm_F<br>RH3_BS_R	GTGGATCATAAAGACTGCCAAAATTC<br>ATAATGCCAACTTTGTACAAGAAAGCTGGGTCGGCGCGCAGTACGGCAATCATATCGAC
	construct	BS_Fin<br>BS_Rin	GGCTATCGCAATTCTGCCTAG<br>TTCCTTTCTCAGTTGGTATGTTATG
	confirmation	ScreenPCR_BSm_F<br>ScreenPCR_BSm_R	CGAATCTGCAGCATGTCTAAA<br>GCTTGAATTTTGGCAGTCTTT
TWW32.1	LH	LH_BS_F<br>LH_BS_R	TTTATAATGCCAACTTTGTACAAAAAAGCAGGCTCCGCGGCTATCGCAATTCTGCCTAG<br>TTAATTCGACCACCGAGGATGGCGTGCGAACCAGTTACTCAGAAAGCTGCCCTTTCCAG
	argB	argB_F<br>argB_R	CTGGTTCGCACGCCATCCTC<br>AGCTTGAAGTATTATGGGATG
	RH	RH1_BS_F<br>RH3_BS_R	CACCTACAGAGTACATCATCCCATAATACTTCAAGCTGGGATCTCCCAAAGAGGCGATC<br>ATAATGCCAACTTTGTACAAGAAAGCTGGGTCGGCGCGCAGTACGGCAATCATATCGAC
	construct	BS_Fin<br>BS_Rin	GGCTATCGCAATTCTGCCTAG<br>TTCCTTTCTCAGTTGGTATGTTATG
	confirmation	ScreenPCR_BSc_F<br>ScreenPCR_BSc_R	AAGCAAGCCGAAACCAAA<br>TTCCTTTCTCAGTTGGTATGTTATG

Primers used for semi-quantitative PCR or quantitative PCR analyses

Strain	Gene	Primer	Sequence (5'-3')
P. expansum strains	*actin*	Semiq_Peactin_F<br>Semiq_Peactin_R	ACATCAAGGAGAAGCTCTGC<br>ATGGATCCACCGATCCAGAC
	PexanA	Semiq_PexanA_F<br>Semiq_PexanA_R	CGGGTTTTGCTGTCGGTC<br>GTTGTCGCAGGCTCGTCT
	PexanB	Semiq_PexanB_F<br>Semiq_PexanB_R	TTCACTTGTCCTGCCACT<br>CAAAACCCATCGTTATCC
	PexanC	Semiq_PexanC_F<br>Semiq_PexanC_R	GCTGTACTATGTTGCGACTG<br>TGAAGGGAACGAAGCCAT
	PexanD	Semiq_PexanD_F<br>Semiq_PexanD_R	GCAGAAAGCGATAACACC<br>CATAAGCGTAGCAGACAGT
	PexanG	Semiq_PexanG_F<br>Semiq_PexanG_R	GAAACAATTCCGCGATAA<br>GAGACGAAGTAGTTCCAAAA
	ctnC	Semiq_ctnC_F<br>Semiq_ctnC_R	GTTGCCTCCTTGTAACCATC<br>CTTCGCCCCATAGAAATAGA
	citS	Semiq_citS_F<br>Semiq_citS_R	GCAAGCGATTCAAGAAGTAT<br>ACCTGGGTTTAGACGCAAGA
	citA	Semiq_citA_F<br>Semiq_citA_R	ACAGGATTGTGGGTGGGACT<br>TGATAGGCAACGAGCGAGAC
	citB	Semiq_citB_F<br>Semiq_citB_R	GCACGCAAAGTAATCAACGA<br>TCTAACACCGCCCAGCACAG
	ctnA	Semiq_ctnA_F<br>Semiq_ctnA_R	GGTTATGGCTGATGGTTGTG<br>GACGCTTTTCCTCTGTCTCG
	citD	Semiq_citD_F<br>Semiq_citD_R	TTCTTCAACAAGGGCGAATC<br>TGACCAATACAACCCCAACG
	citE	Semiq_citE_F<br>Semiq_citE_R	GTAAAGGGCACATACCTCGTG<br>GTCGCCCTGGACAATTTCTT
	citC	Semiq_citC_F<br>Semiq_citC_R	AGATCCTTGAAAACGCACAG<br>AAGCCCATTAGACTAACCGA

***A. fumigatus* strains**	***actin***	**qPCR_Afactin_F**<br>**qPCR_Afactin_R**	**GCGTGAAATTGTTCGTGAC**<br>**GACCTGACCATCGGGAAGT**
	AfxanB	**qPCR_AfxanB_F**<br>**qPCR_AfxanB_R**	**CCTCAAATGGACACGCAGTA**<br>**CGGACATAATCGGCACGGT**
	AfxanC	**Semiq_AfxanC_F**<br>**Semiq_AfxanC_R**	**CCAACCTCTGTCGTTCGTGCTG**<br>**GAGTCTGCCGCGTTTGCTCC**
